# Measurement of longitudinal flow decorrelations in Pb+Pb collisions at $\sqrt{s_{\text {NN}}}=2.76$ and 5.02 TeV with the ATLAS detector

**DOI:** 10.1140/epjc/s10052-018-5605-7

**Published:** 2018-02-19

**Authors:** M. Aaboud, G. Aad, B. Abbott, O. Abdinov, B. Abeloos, S. H. Abidi, O. S. AbouZeid, N. L. Abraham, H. Abramowicz, H. Abreu, R. Abreu, Y. Abulaiti, B. S. Acharya, S. Adachi, L. Adamczyk, J. Adelman, M. Adersberger, T. Adye, A. A. Affolder, Y. Afik, T. Agatonovic-Jovin, C. Agheorghiesei, J. A. Aguilar-Saavedra, S. P. Ahlen, F. Ahmadov, G. Aielli, S. Akatsuka, H. Akerstedt, T. P. A. Åkesson, E. Akilli, A. V. Akimov, G. L. Alberghi, J. Albert, P. Albicocco, M. J. Alconada Verzini, S. C. Alderweireldt, M. Aleksa, I. N. Aleksandrov, C. Alexa, G. Alexander, T. Alexopoulos, M. Alhroob, B. Ali, M. Aliev, G. Alimonti, J. Alison, S. P. Alkire, B. M. M. Allbrooke, B. W. Allen, P. P. Allport, A. Aloisio, A. Alonso, F. Alonso, C. Alpigiani, A. A. Alshehri, M. I. Alstaty, B. Alvarez Gonzalez, D. Álvarez Piqueras, M. G. Alviggi, B. T. Amadio, Y. Amaral Coutinho, C. Amelung, D. Amidei, S. P. Amor Dos Santos, S. Amoroso, G. Amundsen, C. Anastopoulos, L. S. Ancu, N. Andari, T. Andeen, C. F. Anders, J. K. Anders, K. J. Anderson, A. Andreazza, V. Andrei, S. Angelidakis, I. Angelozzi, A. Angerami, A. V. Anisenkov, N. Anjos, A. Annovi, C. Antel, M. Antonelli, A. Antonov, D. J. Antrim, F. Anulli, M. Aoki, L. Aperio Bella, G. Arabidze, Y. Arai, J. P. Araque, V. Araujo Ferraz, A. T. H. Arce, R. E. Ardell, F. A. Arduh, J.-F. Arguin, S. Argyropoulos, M. Arik, A. J. Armbruster, L. J. Armitage, O. Arnaez, H. Arnold, M. Arratia, O. Arslan, A. Artamonov, G. Artoni, S. Artz, S. Asai, N. Asbah, A. Ashkenazi, L. Asquith, K. Assamagan, R. Astalos, M. Atkinson, N. B. Atlay, K. Augsten, G. Avolio, B. Axen, M. K. Ayoub, G. Azuelos, A. E. Baas, M. J. Baca, H. Bachacou, K. Bachas, M. Backes, P. Bagnaia, M. Bahmani, H. Bahrasemani, J. T. Baines, M. Bajic, O. K. Baker, P. J. Bakker, E. M. Baldin, P. Balek, F. Balli, W. K. Balunas, E. Banas, A. Bandyopadhyay, Sw. Banerjee, A. A. E. Bannoura, L. Barak, E. L. Barberio, D. Barberis, M. Barbero, T. Barillari, M.-S. Barisits, J. T. Barkeloo, T. Barklow, N. Barlow, S. L. Barnes, B. M. Barnett, R. M. Barnett, Z. Barnovska-Blenessy, A. Baroncelli, G. Barone, A. J. Barr, L. Barranco Navarro, F. Barreiro, J. Barreiro Guimarães da Costa, R. Bartoldus, A. E. Barton, P. Bartos, A. Basalaev, A. Bassalat, R. L. Bates, S. J. Batista, J. R. Batley, M. Battaglia, M. Bauce, F. Bauer, H. S. Bawa, J. B. Beacham, M. D. Beattie, T. Beau, P. H. Beauchemin, P. Bechtle, H. P. Beck, H. C. Beck, K. Becker, M. Becker, C. Becot, A. J. Beddall, A. Beddall, V. A. Bednyakov, M. Bedognetti, C. P. Bee, T. A. Beermann, M. Begalli, M. Begel, J. K. Behr, A. S. Bell, G. Bella, L. Bellagamba, A. Bellerive, M. Bellomo, K. Belotskiy, O. Beltramello, N. L. Belyaev, O. Benary, D. Benchekroun, M. Bender, N. Benekos, Y. Benhammou, E. Benhar Noccioli, J. Benitez, D. P. Benjamin, M. Benoit, J. R. Bensinger, S. Bentvelsen, L. Beresford, M. Beretta, D. Berge, E. Bergeaas Kuutmann, N. Berger, L. J. Bergsten, J. Beringer, S. Berlendis, N. R. Bernard, G. Bernardi, C. Bernius, F. U. Bernlochner, T. Berry, P. Berta, C. Bertella, G. Bertoli, I. A. Bertram, C. Bertsche, G. J. Besjes, O. Bessidskaia Bylund, M. Bessner, N. Besson, A. Bethani, S. Bethke, A. Betti, A. J. Bevan, J. Beyer, R. M. Bianchi, O. Biebel, D. Biedermann, R. Bielski, K. Bierwagen, N. V. Biesuz, M. Biglietti, T. R. V. Billoud, H. Bilokon, M. Bindi, A. Bingul, C. Bini, S. Biondi, T. Bisanz, C. Bittrich, D. M. Bjergaard, J. E. Black, K. M. Black, R. E. Blair, T. Blazek, I. Bloch, C. Blocker, A. Blue, U. Blumenschein, S. Blunier, G. J. Bobbink, V. S. Bobrovnikov, S. S. Bocchetta, A. Bocci, C. Bock, M. Boehler, D. Boerner, D. Bogavac, A. G. Bogdanchikov, C. Bohm, V. Boisvert, P. Bokan, T. Bold, A. S. Boldyrev, A. E. Bolz, M. Bomben, M. Bona, M. Boonekamp, A. Borisov, G. Borissov, J. Bortfeldt, D. Bortoletto, V. Bortolotto, D. Boscherini, M. Bosman, J. D. Bossio Sola, J. Boudreau, E. V. Bouhova-Thacker, D. Boumediene, C. Bourdarios, S. K. Boutle, A. Boveia, J. Boyd, I. R. Boyko, A. J. Bozson, J. Bracinik, A. Brandt, G. Brandt, O. Brandt, F. Braren, U. Bratzler, B. Brau, J. E. Brau, W. D. Breaden Madden, K. Brendlinger, A. J. Brennan, L. Brenner, R. Brenner, S. Bressler, D. L. Briglin, T. M. Bristow, D. Britton, D. Britzger, F. M. Brochu, I. Brock, R. Brock, G. Brooijmans, T. Brooks, W. K. Brooks, J. Brosamer, E. Brost, J. H Broughton, P. A. Bruckman de Renstrom, D. Bruncko, A. Bruni, G. Bruni, L. S. Bruni, S. Bruno, BH Brunt, M. Bruschi, N. Bruscino, P. Bryant, L. Bryngemark, T. Buanes, Q. Buat, P. Buchholz, A. G. Buckley, I. A. Budagov, F. Buehrer, M. K. Bugge, O. Bulekov, D. Bullock, T. J. Burch, S. Burdin, C. D. Burgard, A. M. Burger, B. Burghgrave, K. Burka, S. Burke, I. Burmeister, J. T. P. Burr, D. Büscher, V. Büscher, P. Bussey, J. M. Butler, C. M. Buttar, J. M. Butterworth, P. Butti, W. Buttinger, A. Buzatu, A. R. Buzykaev, S. Cabrera Urbán, D. Caforio, H. Cai, V. M. Cairo, O. Cakir, N. Calace, P. Calafiura, A. Calandri, G. Calderini, P. Calfayan, G. Callea, L. P. Caloba, S. Calvente Lopez, D. Calvet, S. Calvet, T. P. Calvet, R. Camacho Toro, S. Camarda, P. Camarri, D. Cameron, R. Caminal Armadans, C. Camincher, S. Campana, M. Campanelli, A. Camplani, A. Campoverde, V. Canale, M. Cano Bret, J. Cantero, T. Cao, M. D. M. Capeans Garrido, I. Caprini, M. Caprini, M. Capua, R. M. Carbone, R. Cardarelli, F. Cardillo, I. Carli, T. Carli, G. Carlino, B. T. Carlson, L. Carminati, R. M. D. Carney, S. Caron, E. Carquin, S. Carrá, G. D. Carrillo-Montoya, D. Casadei, M. P. Casado, A. F. Casha, M. Casolino, D. W. Casper, R. Castelijn, V. Castillo Gimenez, N. F. Castro, A. Catinaccio, J. R. Catmore, A. Cattai, J. Caudron, V. Cavaliere, E. Cavallaro, D. Cavalli, M. Cavalli-Sforza, V. Cavasinni, E. Celebi, F. Ceradini, L. Cerda Alberich, A. S. Cerqueira, A. Cerri, L. Cerrito, F. Cerutti, A. Cervelli, S. A. Cetin, A. Chafaq, D. Chakraborty, S. K. Chan, W. S. Chan, Y. L. Chan, P. Chang, J. D. Chapman, D. G. Charlton, C. C. Chau, C. A. Chavez Barajas, S. Che, S. Cheatham, A. Chegwidden, S. Chekanov, S. V. Chekulaev, G. A. Chelkov, M. A. Chelstowska, C. Chen, C. Chen, H. Chen, J. Chen, S. Chen, S. Chen, X. Chen, Y. Chen, H. C. Cheng, H. J. Cheng, A. Cheplakov, E. Cheremushkina, R. Cherkaoui El Moursli, E. Cheu, K. Cheung, L. Chevalier, V. Chiarella, G. Chiarelli, G. Chiodini, A. S. Chisholm, A. Chitan, Y. H. Chiu, M. V. Chizhov, K. Choi, A. R. Chomont, S. Chouridou, Y. S. Chow, V. Christodoulou, M. C. Chu, J. Chudoba, A. J. Chuinard, J. J. Chwastowski, L. Chytka, A. K. Ciftci, D. Cinca, V. Cindro, I. A. Cioara, A. Ciocio, F. Cirotto, Z. H. Citron, M. Citterio, M. Ciubancan, A. Clark, B. L. Clark, M. R. Clark, P. J. Clark, R. N. Clarke, C. Clement, Y. Coadou, M. Cobal, A. Coccaro, J. Cochran, L. Colasurdo, B. Cole, A. P. Colijn, J. Collot, T. Colombo, P. Conde Muiño, E. Coniavitis, S. H. Connell, I. A. Connelly, S. Constantinescu, G. Conti, F. Conventi, M. Cooke, A. M. Cooper-Sarkar, F. Cormier, K. J. R. Cormier, M. Corradi, F. Corriveau, A. Cortes-Gonzalez, G. Costa, M. J. Costa, D. Costanzo, G. Cottin, G. Cowan, B. E. Cox, K. Cranmer, S. J. Crawley, R. A. Creager, G. Cree, S. Crépé-Renaudin, F. Crescioli, W. A. Cribbs, M. Cristinziani, V. Croft, G. Crosetti, A. Cueto, T. Cuhadar Donszelmann, A. R. Cukierman, J. Cummings, M. Curatolo, J. Cúth, S. Czekierda, P. Czodrowski, G. D’amen, S. D’Auria, L. D’eramo, M. D’Onofrio, M. J. Da Cunha Sargedas De Sousa, C. Da Via, W. Dabrowski, T. Dado, T. Dai, O. Dale, F. Dallaire, C. Dallapiccola, M. Dam, J. R. Dandoy, M. F. Daneri, N. P. Dang, A. C. Daniells, N. S. Dann, M. Danninger, M. Dano Hoffmann, V. Dao, G. Darbo, S. Darmora, J. Dassoulas, A. Dattagupta, T. Daubney, W. Davey, C. David, T. Davidek, D. R. Davis, P. Davison, E. Dawe, I. Dawson, K. De, R. de Asmundis, A. De Benedetti, S. De Castro, S. De Cecco, N. De Groot, P. de Jong, H. De la Torre, F. De Lorenzi, A. De Maria, D. De Pedis, A. De Salvo, U. De Sanctis, A. De Santo, K. De Vasconcelos Corga, J. B. De Vivie De Regie, R. Debbe, C. Debenedetti, D. V. Dedovich, N. Dehghanian, I. Deigaard, M. Del Gaudio, J. Del Peso, D. Delgove, F. Deliot, C. M. Delitzsch, A. Dell’Acqua, L. Dell’Asta, M. Dell’Orso, M. Della Pietra, D. della Volpe, M. Delmastro, C. Delporte, P. A. Delsart, D. A. DeMarco, S. Demers, M. Demichev, A. Demilly, S. P. Denisov, D. Denysiuk, D. Derendarz, J. E. Derkaoui, F. Derue, P. Dervan, K. Desch, C. Deterre, K. Dette, M. R. Devesa, P. O. Deviveiros, A. Dewhurst, S. Dhaliwal, F. A. Di Bello, A. Di Ciaccio, L. Di Ciaccio, W. K. Di Clemente, C. Di Donato, A. Di Girolamo, B. Di Girolamo, B. Di Micco, R. Di Nardo, K. F. Di Petrillo, A. Di Simone, R. Di Sipio, D. Di Valentino, C. Diaconu, M. Diamond, F. A. Dias, M. A. Diaz, E. B. Diehl, J. Dietrich, S. Díez Cornell, A. Dimitrievska, J. Dingfelder, P. Dita, S. Dita, F. Dittus, F. Djama, T. Djobava, J. I. Djuvsland, M. A. B. do Vale, D. Dobos, M. Dobre, D. Dodsworth, C. Doglioni, J. Dolejsi, Z. Dolezal, M. Donadelli, S. Donati, P. Dondero, J. Donini, J. Dopke, A. Doria, M. T. Dova, A. T. Doyle, E. Drechsler, M. Dris, Y. Du, J. Duarte-Campderros, F. Dubinin, A. Dubreuil, E. Duchovni, G. Duckeck, A. Ducourthial, O. A. Ducu, D. Duda, A. Dudarev, A. Chr. Dudder, E. M. Duffield, L. Duflot, M. Dührssen, C. Dulsen, M. Dumancic, A. E. Dumitriu, A. K. Duncan, M. Dunford, A. Duperrin, H. Duran Yildiz, M. Düren, A. Durglishvili, D. Duschinger, B. Dutta, D. Duvnjak, M. Dyndal, B. S. Dziedzic, C. Eckardt, K. M. Ecker, R. C. Edgar, T. Eifert, G. Eigen, K. Einsweiler, T. Ekelof, M. El Kacimi, R. El Kosseifi, V. Ellajosyula, M. Ellert, S. Elles, F. Ellinghaus, A. A. Elliot, N. Ellis, J. Elmsheuser, M. Elsing, D. Emeliyanov, Y. Enari, J. S. Ennis, M. B. Epland, J. Erdmann, A. Ereditato, M. Ernst, S. Errede, M. Escalier, C. Escobar, B. Esposito, O. Estrada Pastor, A. I. Etienvre, E. Etzion, H. Evans, A. Ezhilov, M. Ezzi, F. Fabbri, L. Fabbri, V. Fabiani, G. Facini, R. M. Fakhrutdinov, S. Falciano, R. J. Falla, J. Faltova, Y. Fang, M. Fanti, A. Farbin, A. Farilla, C. Farina, E. M. Farina, T. Farooque, S. Farrell, S. M. Farrington, P. Farthouat, F. Fassi, P. Fassnacht, D. Fassouliotis, M. Faucci Giannelli, A. Favareto, W. J. Fawcett, L. Fayard, O. L. Fedin, W. Fedorko, S. Feigl, L. Feligioni, C. Feng, E. J. Feng, M. J. Fenton, A. B. Fenyuk, L. Feremenga, P. Fernandez Martinez, J. Ferrando, A. Ferrari, P. Ferrari, R. Ferrari, D. E. Ferreira de Lima, A. Ferrer, D. Ferrere, C. Ferretti, F. Fiedler, A. Filipčič, M. Filipuzzi, F. Filthaut, M. Fincke-Keeler, K. D. Finelli, M. C. N. Fiolhais, L. Fiorini, A. Fischer, C. Fischer, J. Fischer, W. C. Fisher, N. Flaschel, I. Fleck, P. Fleischmann, R. R. M. Fletcher, T. Flick, B. M. Flierl, L. R. Flores Castillo, M. J. Flowerdew, G. T. Forcolin, A. Formica, F. A. Förster, A. Forti, A. G. Foster, D. Fournier, H. Fox, S. Fracchia, P. Francavilla, M. Franchini, S. Franchino, D. Francis, L. Franconi, M. Franklin, M. Frate, M. Fraternali, D. Freeborn, S. M. Fressard-Batraneanu, B. Freund, D. Froidevaux, J. A. Frost, C. Fukunaga, T. Fusayasu, J. Fuster, O. Gabizon, A. Gabrielli, A. Gabrielli, G. P. Gach, S. Gadatsch, S. Gadomski, G. Gagliardi, L. G. Gagnon, C. Galea, B. Galhardo, E. J. Gallas, B. J. Gallop, P. Gallus, G. Galster, K. K. Gan, S. Ganguly, Y. Gao, Y. S. Gao, F. M. Garay Walls, C. García, J. E. García Navarro, J. A. García Pascual, M. Garcia-Sciveres, R. W. Gardner, N. Garelli, V. Garonne, A. Gascon Bravo, K. Gasnikova, C. Gatti, A. Gaudiello, G. Gaudio, I. L. Gavrilenko, C. Gay, G. Gaycken, E. N. Gazis, C. N. P. Gee, J. Geisen, M. Geisen, M. P. Geisler, K. Gellerstedt, C. Gemme, M. H. Genest, C. Geng, S. Gentile, C. Gentsos, S. George, D. Gerbaudo, G. Geßner, S. Ghasemi, M. Ghneimat, B. Giacobbe, S. Giagu, N. Giangiacomi, P. Giannetti, S. M. Gibson, M. Gignac, M. Gilchriese, D. Gillberg, G. Gilles, D. M. Gingrich, M. P. Giordani, F. M. Giorgi, P. F. Giraud, P. Giromini, G. Giugliarelli, D. Giugni, F. Giuli, C. Giuliani, M. Giulini, B. K. Gjelsten, S. Gkaitatzis, I. Gkialas, E. L. Gkougkousis, P. Gkountoumis, L. K. Gladilin, C. Glasman, J. Glatzer, P. C. F. Glaysher, A. Glazov, M. Goblirsch-Kolb, J. Godlewski, S. Goldfarb, T. Golling, D. Golubkov, A. Gomes, R. Gonçalo, R. Goncalves Gama, J. Goncalves Pinto Firmino Da Costa, G. Gonella, L. Gonella, A. Gongadze, J. L. Gonski, S. González de la Hoz, S. Gonzalez-Sevilla, L. Goossens, P. A. Gorbounov, H. A. Gordon, I. Gorelov, B. Gorini, E. Gorini, A. Gorišek, A. T. Goshaw, C. Gössling, M. I. Gostkin, C. A. Gottardo, C. R. Goudet, D. Goujdami, A. G. Goussiou, N. Govender, E. Gozani, I. Grabowska-Bold, P. O. J. Gradin, J. Gramling, E. Gramstad, S. Grancagnolo, V. Gratchev, P. M. Gravila, C. Gray, H. M. Gray, Z. D. Greenwood, C. Grefe, K. Gregersen, I. M. Gregor, P. Grenier, K. Grevtsov, J. Griffiths, A. A. Grillo, K. Grimm, S. Grinstein, Ph. Gris, J.-F. Grivaz, S. Groh, E. Gross, J. Grosse-Knetter, G. C. Grossi, Z. J. Grout, A. Grummer, L. Guan, W. Guan, J. Guenther, F. Guescini, D. Guest, O. Gueta, B. Gui, E. Guido, T. Guillemin, S. Guindon, U. Gul, C. Gumpert, J. Guo, W. Guo, Y. Guo, R. Gupta, S. Gurbuz, G. Gustavino, B. J. Gutelman, P. Gutierrez, N. G. Gutierrez Ortiz, C. Gutschow, C. Guyot, M. P. Guzik, C. Gwenlan, C. B. Gwilliam, A. Haas, C. Haber, H. K. Hadavand, N. Haddad, A. Hadef, S. Hageböck, M. Hagihara, H. Hakobyan, M. Haleem, J. Haley, G. Halladjian, G. D. Hallewell, K. Hamacher, P. Hamal, K. Hamano, A. Hamilton, G. N. Hamity, P. G. Hamnett, L. Han, S. Han, K. Hanagaki, K. Hanawa, M. Hance, D. M. Handl, B. Haney, P. Hanke, J. B. Hansen, J. D. Hansen, M. C. Hansen, P. H. Hansen, K. Hara, A. S. Hard, T. Harenberg, F. Hariri, S. Harkusha, P. F. Harrison, N. M. Hartmann, Y. Hasegawa, A. Hasib, S. Hassani, S. Haug, R. Hauser, L. Hauswald, L. B. Havener, M. Havranek, C. M. Hawkes, R. J. Hawkings, D. Hayakawa, D. Hayden, C. P. Hays, J. M. Hays, H. S. Hayward, S. J. Haywood, S. J. Head, T. Heck, V. Hedberg, L. Heelan, S. Heer, K. K. Heidegger, S. Heim, T. Heim, B. Heinemann, J. J. Heinrich, L. Heinrich, C. Heinz, J. Hejbal, L. Helary, A. Held, S. Hellman, C. Helsens, R. C. W. Henderson, Y. Heng, S. Henkelmann, A. M. Henriques Correia, S. Henrot-Versille, G. H. Herbert, H. Herde, V. Herget, Y. Hernández Jiménez, H. Herr, G. Herten, R. Hertenberger, L. Hervas, T. C. Herwig, G. G. Hesketh, N. P. Hessey, J. W. Hetherly, S. Higashino, E. Higón-Rodriguez, K. Hildebrand, E. Hill, J. C. Hill, K. H. Hiller, S. J. Hillier, M. Hils, I. Hinchliffe, M. Hirose, D. Hirschbuehl, B. Hiti, O. Hladik, D. R. Hlaluku, X. Hoad, J. Hobbs, N. Hod, M. C. Hodgkinson, P. Hodgson, A. Hoecker, M. R. Hoeferkamp, F. Hoenig, D. Hohn, T. R. Holmes, M. Homann, S. Honda, T. Honda, T. M. Hong, B. H. Hooberman, W. H. Hopkins, Y. Horii, A. J. Horton, J-Y. Hostachy, A. Hostiuc, S. Hou, A. Hoummada, J. Howarth, J. Hoya, M. Hrabovsky, J. Hrdinka, I. Hristova, J. Hrivnac, T. Hryn’ova, A. Hrynevich, P. J. Hsu, S.-C. Hsu, Q. Hu, S. Hu, Y. Huang, Z. Hubacek, F. Hubaut, F. Huegging, T. B. Huffman, E. W. Hughes, M. Huhtinen, R. F. H. Hunter, P. Huo, N. Huseynov, J. Huston, J. Huth, R. Hyneman, G. Iacobucci, G. Iakovidis, I. Ibragimov, L. Iconomidou-Fayard, Z. Idrissi, P. Iengo, O. Igonkina, T. Iizawa, Y. Ikegami, M. Ikeno, Y. Ilchenko, D. Iliadis, N. Ilic, F. Iltzsche, G. Introzzi, P. Ioannou, M. Iodice, K. Iordanidou, V. Ippolito, M. F. Isacson, N. Ishijima, M. Ishino, M. Ishitsuka, C. Issever, S. Istin, F. Ito, J. M. Iturbe Ponce, R. Iuppa, H. Iwasaki, J. M. Izen, V. Izzo, S. Jabbar, P. Jackson, R. M. Jacobs, V. Jain, K. B. Jakobi, K. Jakobs, S. Jakobsen, T. Jakoubek, D. O. Jamin, D. K. Jana, R. Jansky, J. Janssen, M. Janus, P. A. Janus, G. Jarlskog, N. Javadov, T. Javůrek, M. Javurkova, F. Jeanneau, L. Jeanty, J. Jejelava, A. Jelinskas, P. Jenni, C. Jeske, S. Jézéquel, H. Ji, J. Jia, H. Jiang, Y. Jiang, Z. Jiang, S. Jiggins, J. Jimenez Pena, S. Jin, A. Jinaru, O. Jinnouchi, H. Jivan, P. Johansson, K. A. Johns, C. A. Johnson, W. J. Johnson, K. Jon-And, R. W. L. Jones, S. D. Jones, S. Jones, T. J. Jones, J. Jongmanns, P. M. Jorge, J. Jovicevic, X. Ju, A. Juste Rozas, M. K. Köhler, A. Kaczmarska, M. Kado, H. Kagan, M. Kagan, S. J. Kahn, T. Kaji, E. Kajomovitz, C. W. Kalderon, A. Kaluza, S. Kama, A. Kamenshchikov, N. Kanaya, L. Kanjir, V. A. Kantserov, J. Kanzaki, B. Kaplan, L. S. Kaplan, D. Kar, K. Karakostas, N. Karastathis, M. J. Kareem, E. Karentzos, S. N. Karpov, Z. M. Karpova, K. Karthik, V. Kartvelishvili, A. N. Karyukhin, K. Kasahara, L. Kashif, R. D. Kass, A. Kastanas, Y. Kataoka, C. Kato, A. Katre, J. Katzy, K. Kawade, K. Kawagoe, T. Kawamoto, G. Kawamura, E. F. Kay, V. F. Kazanin, R. Keeler, R. Kehoe, J. S. Keller, E. Kellermann, J. J. Kempster, J Kendrick, H. Keoshkerian, O. Kepka, B. P. Kerševan, S. Kersten, R. A. Keyes, M. Khader, F. Khalil-zada, A. Khanov, A. G. Kharlamov, T. Kharlamova, A. Khodinov, T. J. Khoo, V. Khovanskiy, E. Khramov, J. Khubua, S. Kido, C. R. Kilby, H. Y. Kim, S. H. Kim, Y. K. Kim, N. Kimura, O. M. Kind, B. T. King, D. Kirchmeier, J. Kirk, A. E. Kiryunin, T. Kishimoto, D. Kisielewska, V. Kitali, O. Kivernyk, E. Kladiva, T. Klapdor-Kleingrothaus, M. H. Klein, M. Klein, U. Klein, K. Kleinknecht, P. Klimek, A. Klimentov, R. Klingenberg, T. Klingl, T. Klioutchnikova, F. F. Klitzner, E.-E. Kluge, P. Kluit, S. Kluth, E. Kneringer, E. B. F. G. Knoops, A. Knue, A. Kobayashi, D. Kobayashi, T. Kobayashi, M. Kobel, M. Kocian, P. Kodys, T. Koffas, E. Koffeman, N. M. Köhler, T. Koi, M. Kolb, I. Koletsou, A. A. Komar, T. Kondo, N. Kondrashova, K. Köneke, A. C. König, T. Kono, R. Konoplich, N. Konstantinidis, B. Konya, R. Kopeliansky, S. Koperny, A. K. Kopp, K. Korcyl, K. Kordas, A. Korn, A. A. Korol, I. Korolkov, E. V. Korolkova, O. Kortner, S. Kortner, T. Kosek, V. V. Kostyukhin, A. Kotwal, A. Koulouris, A. Kourkoumeli-Charalampidi, C. Kourkoumelis, E. Kourlitis, V. Kouskoura, A. B. Kowalewska, R. Kowalewski, T. Z. Kowalski, C. Kozakai, W. Kozanecki, A. S. Kozhin, V. A. Kramarenko, G. Kramberger, D. Krasnopevtsev, M. W. Krasny, A. Krasznahorkay, D. Krauss, J. A. Kremer, J. Kretzschmar, K. Kreutzfeldt, P. Krieger, K. Krizka, K. Kroeninger, H. Kroha, J. Kroll, J. Kroll, J. Kroseberg, J. Krstic, U. Kruchonak, H. Krüger, N. Krumnack, M. C. Kruse, T. Kubota, H. Kucuk, S. Kuday, J. T. Kuechler, S. Kuehn, A. Kugel, F. Kuger, T. Kuhl, V. Kukhtin, R. Kukla, Y. Kulchitsky, S. Kuleshov, Y. P. Kulinich, M. Kuna, T. Kunigo, A. Kupco, T. Kupfer, O. Kuprash, H. Kurashige, L. L. Kurchaninov, Y. A. Kurochkin, M. G. Kurth, E. S. Kuwertz, M. Kuze, J. Kvita, T. Kwan, D. Kyriazopoulos, A. La Rosa, J. L. La Rosa Navarro, L. La Rotonda, F. La Ruffa, C. Lacasta, F. Lacava, J. Lacey, D. P. J. Lack, H. Lacker, D. Lacour, E. Ladygin, R. Lafaye, B. Laforge, T. Lagouri, S. Lai, S. Lammers, W. Lampl, E. Lançon, U. Landgraf, M. P. J. Landon, M. C. Lanfermann, V. S. Lang, J. C. Lange, R. J. Langenberg, A. J. Lankford, F. Lanni, K. Lantzsch, A. Lanza, A. Lapertosa, S. Laplace, J. F. Laporte, T. Lari, F. Lasagni Manghi, M. Lassnig, T. S. Lau, P. Laurelli, W. Lavrijsen, A. T. Law, P. Laycock, T. Lazovich, M. Lazzaroni, B. Le, O. Le Dortz, E. Le Guirriec, E. P. Le Quilleuc, M. LeBlanc, T. LeCompte, F. Ledroit-Guillon, C. A. Lee, G. R. Lee, S. C. Lee, L. Lee, B. Lefebvre, G. Lefebvre, M. Lefebvre, F. Legger, C. Leggett, G. Lehmann Miotto, X. Lei, W. A. Leight, M. A. L. Leite, R. Leitner, D. Lellouch, B. Lemmer, K. J. C. Leney, T. Lenz, B. Lenzi, R. Leone, S. Leone, C. Leonidopoulos, G. Lerner, C. Leroy, R. Les, A. A. J. Lesage, C. G. Lester, M. Levchenko, J. Levêque, D. Levin, L. J. Levinson, M. Levy, D. Lewis, B. Li, Changqiao Li, H. Li, L. Li, Q. Li, Q. Li, S. Li, X. Li, Y. Li, Z. Liang, B. Liberti, A. Liblong, K. Lie, J. Liebal, W. Liebig, A. Limosani, C. Y. Lin, K. Lin, S. C. Lin, T. H. Lin, R. A. Linck, B. E. Lindquist, A. E. Lionti, E. Lipeles, A. Lipniacka, M. Lisovyi, T. M. Liss, A. Lister, A. M. Litke, B. Liu, H. Liu, H. Liu, J. K. K. Liu, J. Liu, J. B. Liu, K. Liu, L. Liu, M. Liu, Y. L. Liu, Y. Liu, M. Livan, A. Lleres, J. Llorente Merino, S. L. Lloyd, C. Y. Lo, F. Lo Sterzo, E. M. Lobodzinska, P. Loch, F. K. Loebinger, A. Loesle, K. M. Loew, T. Lohse, K. Lohwasser, M. Lokajicek, B. A. Long, J. D. Long, R. E. Long, L. Longo, K. A. Looper, J. A. Lopez, I. Lopez Paz, A. Lopez Solis, J. Lorenz, N. Lorenzo Martinez, M. Losada, P. J. Lösel, X. Lou, A. Lounis, J. Love, P. A. Love, H. Lu, N. Lu, Y. J. Lu, H. J. Lubatti, C. Luci, A. Lucotte, C. Luedtke, F. Luehring, W. Lukas, L. Luminari, O. Lundberg, B. Lund-Jensen, M. S. Lutz, P. M. Luzi, D. Lynn, R. Lysak, E. Lytken, F. Lyu, V. Lyubushkin, H. Ma, L. L. Ma, Y. Ma, G. Maccarrone, A. Macchiolo, C. M. Macdonald, B. Maček, J. Machado Miguens, D. Madaffari, R. Madar, W. F. Mader, A. Madsen, N. Madysa, J. Maeda, S. Maeland, T. Maeno, A. S. Maevskiy, V. Magerl, C. Maiani, C. Maidantchik, T. Maier, A. Maio, O. Majersky, S. Majewski, Y. Makida, N. Makovec, B. Malaescu, Pa. Malecki, V. P. Maleev, F. Malek, U. Mallik, D. Malon, C. Malone, S. Maltezos, S. Malyukov, J. Mamuzic, G. Mancini, I. Mandić, J. Maneira, L. Manhaes de Andrade Filho, J. Manjarres Ramos, K. H. Mankinen, A. Mann, A. Manousos, B. Mansoulie, J. D. Mansour, R. Mantifel, M. Mantoani, S. Manzoni, L. Mapelli, G. Marceca, L. March, L. Marchese, G. Marchiori, M. Marcisovsky, C. A. Marin Tobon, M. Marjanovic, D. E. Marley, F. Marroquim, S. P. Marsden, Z. Marshall, M. U. F Martensson, S. Marti-Garcia, C. B. Martin, T. A. Martin, V. J. Martin, B. Martin dit Latour, M. Martinez, V. I. Martinez Outschoorn, S. Martin-Haugh, V. S. Martoiu, A. C. Martyniuk, A. Marzin, L. Masetti, T. Mashimo, R. Mashinistov, J. Masik, A. L. Maslennikov, L. H. Mason, L. Massa, P. Mastrandrea, A. Mastroberardino, T. Masubuchi, P. Mättig, J. Maurer, S. J. Maxfield, D. A. Maximov, R. Mazini, I. Maznas, S. M. Mazza, N. C. Mc Fadden, G. Mc Goldrick, S. P. Mc Kee, A. McCarn, R. L. McCarthy, T. G. McCarthy, L. I. McClymont, E. F. McDonald, J. A. Mcfayden, G. Mchedlidze, S. J. McMahon, P. C. McNamara, C. J. McNicol, R. A. McPherson, S. Meehan, T. J. Megy, S. Mehlhase, A. Mehta, T. Meideck, K. Meier, B. Meirose, D. Melini, B. R. Mellado Garcia, J. D. Mellenthin, M. Melo, F. Meloni, A. Melzer, S. B. Menary, L. Meng, X. T. Meng, A. Mengarelli, S. Menke, E. Meoni, S. Mergelmeyer, C. Merlassino, P. Mermod, L. Merola, C. Meroni, F. S. Merritt, A. Messina, J. Metcalfe, A. S. Mete, C. Meyer, J.-P. Meyer, J. Meyer, H. Meyer Zu Theenhausen, F. Miano, R. P. Middleton, S. Miglioranzi, L. Mijović, G. Mikenberg, M. Mikestikova, M. Mikuž, M. Milesi, A. Milic, D. A. Millar, D. W. Miller, C. Mills, A. Milov, D. A. Milstead, A. A. Minaenko, Y. Minami, I. A. Minashvili, A. I. Mincer, B. Mindur, M. Mineev, Y. Minegishi, Y. Ming, L. M. Mir, A. Mirto, K. P. Mistry, T. Mitani, J. Mitrevski, V. A. Mitsou, A. Miucci, P. S. Miyagawa, A. Mizukami, J. U. Mjörnmark, T. Mkrtchyan, M. Mlynarikova, T. Moa, K. Mochizuki, P. Mogg, S. Mohapatra, S. Molander, R. Moles-Valls, M. C. Mondragon, K. Mönig, J. Monk, E. Monnier, A. Montalbano, J. Montejo Berlingen, F. Monticelli, S. Monzani, R. W. Moore, N. Morange, D. Moreno, M. Moreno Llácer, P. Morettini, S. Morgenstern, D. Mori, T. Mori, M. Morii, M. Morinaga, V. Morisbak, A. K. Morley, G. Mornacchi, J. D. Morris, L. Morvaj, P. Moschovakos, M. Mosidze, H. J. Moss, J. Moss, K. Motohashi, R. Mount, E. Mountricha, E. J. W. Moyse, S. Muanza, F. Mueller, J. Mueller, R. S. P. Mueller, D. Muenstermann, P. Mullen, G. A. Mullier, F. J. Munoz Sanchez, W. J. Murray, H. Musheghyan, M. Muškinja, A. G. Myagkov, M. Myska, B. P. Nachman, O. Nackenhorst, K. Nagai, R. Nagai, K. Nagano, Y. Nagasaka, K. Nagata, M. Nagel, E. Nagy, A. M. Nairz, Y. Nakahama, K. Nakamura, T. Nakamura, I. Nakano, R. F. Naranjo Garcia, R. Narayan, D. I. Narrias Villar, I. Naryshkin, T. Naumann, G. Navarro, R. Nayyar, H. A. Neal, P. Yu. Nechaeva, T. J. Neep, A. Negri, M. Negrini, S. Nektarijevic, C. Nellist, A. Nelson, M. E. Nelson, S. Nemecek, P. Nemethy, M. Nessi, M. S. Neubauer, M. Neumann, P. R. Newman, T. Y. Ng, Y. S. Ng, T. Nguyen Manh, R. B. Nickerson, R. Nicolaidou, J. Nielsen, N. Nikiforou, V. Nikolaenko, I. Nikolic-Audit, K. Nikolopoulos, P. Nilsson, Y. Ninomiya, A. Nisati, N. Nishu, R. Nisius, I. Nitsche, T. Nitta, T. Nobe, Y. Noguchi, M. Nomachi, I. Nomidis, M. A. Nomura, T. Nooney, M. Nordberg, N. Norjoharuddeen, O. Novgorodova, M. Nozaki, L. Nozka, K. Ntekas, E. Nurse, F. Nuti, K. O’connor, D. C. O’Neil, A. A. O’Rourke, V. O’Shea, F. G. Oakham, H. Oberlack, T. Obermann, J. Ocariz, A. Ochi, I. Ochoa, J. P. Ochoa-Ricoux, S. Oda, S. Odaka, A. Oh, S. H. Oh, C. C. Ohm, H. Ohman, H. Oide, H. Okawa, Y. Okumura, T. Okuyama, A. Olariu, L. F. Oleiro Seabra, S. A. Olivares Pino, D. Oliveira Damazio, M. J. R. Olsson, A. Olszewski, J. Olszowska, A. Onofre, K. Onogi, P. U. E. Onyisi, H. Oppen, M. J. Oreglia, Y. Oren, D. Orestano, N. Orlando, R. S. Orr, B. Osculati, R. Ospanov, G. Otero y Garzon, H. Otono, M. Ouchrif, F. Ould-Saada, A. Ouraou, K. P. Oussoren, Q. Ouyang, M. Owen, R. E. Owen, V. E. Ozcan, N. Ozturk, K. Pachal, A. Pacheco Pages, L. Pacheco Rodriguez, C. Padilla Aranda, S. Pagan Griso, M. Paganini, F. Paige, G. Palacino, S. Palazzo, S. Palestini, M. Palka, D. Pallin, E. St. Panagiotopoulou, I. Panagoulias, C. E. Pandini, J. G. Panduro Vazquez, P. Pani, S. Panitkin, D. Pantea, L. Paolozzi, Th. D. Papadopoulou, K. Papageorgiou, A. Paramonov, D. Paredes Hernandez, A. J. Parker, M. A. Parker, K. A. Parker, F. Parodi, J. A. Parsons, U. Parzefall, V. R. Pascuzzi, J. M. Pasner, E. Pasqualucci, S. Passaggio, Fr. Pastore, S. Pataraia, J. R. Pater, T. Pauly, B. Pearson, S. Pedraza Lopez, R. Pedro, S. V. Peleganchuk, O. Penc, C. Peng, H. Peng, J. Penwell, B. S. Peralva, M. M. Perego, D. V. Perepelitsa, F. Peri, L. Perini, H. Pernegger, S. Perrella, R. Peschke, V. D. Peshekhonov, K. Peters, R. F. Y. Peters, B. A. Petersen, T. C. Petersen, E. Petit, A. Petridis, C. Petridou, P. Petroff, E. Petrolo, M. Petrov, F. Petrucci, N. E. Pettersson, A. Peyaud, R. Pezoa, F. H. Phillips, P. W. Phillips, G. Piacquadio, E. Pianori, A. Picazio, M. A. Pickering, R. Piegaia, J. E. Pilcher, A. D. Pilkington, M. Pinamonti, J. L. Pinfold, H. Pirumov, M. Pitt, L. Plazak, M.-A. Pleier, V. Pleskot, E. Plotnikova, D. Pluth, P. Podberezko, R. Poettgen, R. Poggi, L. Poggioli, I. Pogrebnyak, D. Pohl, I. Pokharel, G. Polesello, A. Poley, A. Policicchio, R. Polifka, A. Polini, C. S. Pollard, V. Polychronakos, K. Pommès, D. Ponomarenko, L. Pontecorvo, G. A. Popeneciu, D. M. Portillo Quintero, S. Pospisil, K. Potamianos, I. N. Potrap, C. J. Potter, H. Potti, T. Poulsen, J. Poveda, M. E. Pozo Astigarraga, P. Pralavorio, A. Pranko, S. Prell, D. Price, M. Primavera, S. Prince, N. Proklova, K. Prokofiev, F. Prokoshin, S. Protopopescu, J. Proudfoot, M. Przybycien, A. Puri, P. Puzo, J. Qian, G. Qin, Y. Qin, A. Quadt, M. Queitsch-Maitland, D. Quilty, S. Raddum, V. Radeka, V. Radescu, S. K. Radhakrishnan, P. Radloff, P. Rados, F. Ragusa, G. Rahal, J. A. Raine, S. Rajagopalan, C. Rangel-Smith, T. Rashid, S. Raspopov, M. G. Ratti, D. M. Rauch, F. Rauscher, S. Rave, I. Ravinovich, J. H. Rawling, M. Raymond, A. L. Read, N. P. Readioff, M. Reale, D. M. Rebuzzi, A. Redelbach, G. Redlinger, R. Reece, R. G. Reed, K. Reeves, L. Rehnisch, J. Reichert, A. Reiss, C. Rembser, H. Ren, M. Rescigno, S. Resconi, E. D. Resseguie, S. Rettie, E. Reynolds, O. L. Rezanova, P. Reznicek, R. Rezvani, R. Richter, S. Richter, E. Richter-Was, O. Ricken, M. Ridel, P. Rieck, C. J. Riegel, J. Rieger, O. Rifki, M. Rijssenbeek, A. Rimoldi, M. Rimoldi, L. Rinaldi, G. Ripellino, B. Ristić, E. Ritsch, I. Riu, F. Rizatdinova, E. Rizvi, C. Rizzi, R. T. Roberts, S. H. Robertson, A. Robichaud-Veronneau, D. Robinson, J. E. M. Robinson, A. Robson, E. Rocco, C. Roda, Y. Rodina, S. Rodriguez Bosca, A. Rodriguez Perez, D. Rodriguez Rodriguez, S. Roe, C. S. Rogan, O. Røhne, J. Roloff, A. Romaniouk, M. Romano, S. M. Romano Saez, E. Romero Adam, N. Rompotis, M. Ronzani, L. Roos, S. Rosati, K. Rosbach, P. Rose, N.-A. Rosien, E. Rossi, L. P. Rossi, J. H. N. Rosten, R. Rosten, M. Rotaru, J. Rothberg, D. Rousseau, A. Rozanov, Y. Rozen, X. Ruan, F. Rubbo, E. M. Ruettinger, F. Rühr, A. Ruiz-Martinez, Z. Rurikova, N. A. Rusakovich, H. L. Russell, J. P. Rutherfoord, N. Ruthmann, Y. F. Ryabov, M. Rybar, G. Rybkin, S. Ryu, A. Ryzhov, G. F. Rzehorz, A. F. Saavedra, G. Sabato, S. Sacerdoti, H. F.-W. Sadrozinski, R. Sadykov, F. Safai Tehrani, P. Saha, M. Sahinsoy, M. Saimpert, M. Saito, T. Saito, H. Sakamoto, Y. Sakurai, G. Salamanna, J. E. Salazar Loyola, D. Salek, P. H. Sales De Bruin, D. Salihagic, A. Salnikov, J. Salt, D. Salvatore, F. Salvatore, A. Salvucci, A. Salzburger, D. Sammel, D. Sampsonidis, D. Sampsonidou, J. Sánchez, V. Sanchez Martinez, A. Sanchez Pineda, H. Sandaker, R. L. Sandbach, C. O. Sander, M. Sandhoff, C. Sandoval, D. P. C. Sankey, M. Sannino, Y. Sano, A. Sansoni, C. Santoni, H. Santos, I. Santoyo Castillo, A. Sapronov, J. G. Saraiva, B. Sarrazin, O. Sasaki, K. Sato, E. Sauvan, G. Savage, P. Savard, N. Savic, C. Sawyer, L. Sawyer, J. Saxon, C. Sbarra, A. Sbrizzi, T. Scanlon, D. A. Scannicchio, J. Schaarschmidt, P. Schacht, B. M. Schachtner, D. Schaefer, L. Schaefer, R. Schaefer, J. Schaeffer, S. Schaepe, S. Schaetzel, U. Schäfer, A. C. Schaffer, D. Schaile, R. D. Schamberger, V. A. Schegelsky, D. Scheirich, M. Schernau, C. Schiavi, S. Schier, L. K. Schildgen, C. Schillo, M. Schioppa, S. Schlenker, K. R. Schmidt-Sommerfeld, K. Schmieden, C. Schmitt, S. Schmitt, S. Schmitz, U. Schnoor, L. Schoeffel, A. Schoening, B. D. Schoenrock, E. Schopf, M. Schott, J. F. P. Schouwenberg, J. Schovancova, S. Schramm, N. Schuh, A. Schulte, M. J. Schultens, H.-C. Schultz-Coulon, H. Schulz, M. Schumacher, B. A. Schumm, Ph. Schune, A. Schwartzman, T. A. Schwarz, H. Schweiger, Ph. Schwemling, R. Schwienhorst, J. Schwindling, A. Sciandra, G. Sciolla, M. Scornajenghi, F. Scuri, F. Scutti, J. Searcy, P. Seema, S. C. Seidel, A. Seiden, J. M. Seixas, G. Sekhniaidze, K. Sekhon, S. J. Sekula, N. Semprini-Cesari, S. Senkin, C. Serfon, L. Serin, L. Serkin, M. Sessa, R. Seuster, H. Severini, T. Sfiligoj, F. Sforza, A. Sfyrla, E. Shabalina, N. W. Shaikh, L. Y. Shan, R. Shang, J. T. Shank, M. Shapiro, P. B. Shatalov, K. Shaw, S. M. Shaw, A. Shcherbakova, C. Y. Shehu, Y. Shen, N. Sherafati, A. D. Sherman, P. Sherwood, L. Shi, S. Shimizu, C. O. Shimmin, M. Shimojima, I. P. J. Shipsey, S. Shirabe, M. Shiyakova, J. Shlomi, A. Shmeleva, D. Shoaleh Saadi, M. J. Shochet, S. Shojaii, D. R. Shope, S. Shrestha, E. Shulga, M. A. Shupe, P. Sicho, A. M. Sickles, P. E. Sidebo, E. Sideras Haddad, O. Sidiropoulou, A. Sidoti, F. Siegert, Dj. Sijacki, J. Silva, S. B. Silverstein, V. Simak, L. Simic, S. Simion, E. Simioni, B. Simmons, M. Simon, P. Sinervo, N. B. Sinev, M. Sioli, G. Siragusa, I. Siral, S. Yu. Sivoklokov, J. Sjölin, M. B. Skinner, P. Skubic, M. Slater, T. Slavicek, M. Slawinska, K. Sliwa, R. Slovak, V. Smakhtin, B. H. Smart, J. Smiesko, N. Smirnov, S. Yu. Smirnov, Y. Smirnov, L. N. Smirnova, O. Smirnova, J. W. Smith, M. N. K. Smith, R. W. Smith, M. Smizanska, K. Smolek, A. A. Snesarev, I. M. Snyder, S. Snyder, R. Sobie, F. Socher, A. Soffer, A. Søgaard, D. A. Soh, G. Sokhrannyi, C. A. Solans Sanchez, M. Solar, E. Yu. Soldatov, U. Soldevila, A. A. Solodkov, A. Soloshenko, O. V. Solovyanov, V. Solovyev, P. Sommer, H. Son, A. Sopczak, D. Sosa, C. L. Sotiropoulou, S. Sottocornola, R. Soualah, A. M. Soukharev, D. South, B. C. Sowden, S. Spagnolo, M. Spalla, M. Spangenberg, F. Spanò, D. Sperlich, F. Spettel, T. M. Spieker, R. Spighi, G. Spigo, L. A. Spiller, M. Spousta, R. D. St. Denis, A. Stabile, R. Stamen, S. Stamm, E. Stanecka, R. W. Stanek, C. Stanescu, M. M. Stanitzki, B. S. Stapf, S. Stapnes, E. A. Starchenko, G. H. Stark, J. Stark, S. H Stark, P. Staroba, P. Starovoitov, S. Stärz, R. Staszewski, M. Stegler, P. Steinberg, B. Stelzer, H. J. Stelzer, O. Stelzer-Chilton, H. Stenzel, T. J. Stevenson, G. A. Stewart, M. C. Stockton, M. Stoebe, G. Stoicea, P. Stolte, S. Stonjek, A. R. Stradling, A. Straessner, M. E. Stramaglia, J. Strandberg, S. Strandberg, M. Strauss, P. Strizenec, R. Ströhmer, D. M. Strom, R. Stroynowski, A. Strubig, S. A. Stucci, B. Stugu, N. A. Styles, D. Su, J. Su, S. Suchek, Y. Sugaya, M. Suk, V. V. Sulin, D. M. S. Sultan, S. Sultansoy, T. Sumida, S. Sun, X. Sun, K. Suruliz, C. J. E. Suster, M. R. Sutton, S. Suzuki, M. Svatos, M. Swiatlowski, S. P. Swift, I. Sykora, T. Sykora, D. Ta, K. Tackmann, J. Taenzer, A. Taffard, R. Tafirout, E. Tahirovic, N. Taiblum, H. Takai, R. Takashima, E. H. Takasugi, K. Takeda, T. Takeshita, Y. Takubo, M. Talby, A. A. Talyshev, J. Tanaka, M. Tanaka, R. Tanaka, S. Tanaka, R. Tanioka, B. B. Tannenwald, S. Tapia Araya, S. Tapprogge, S. Tarem, G. F. Tartarelli, P. Tas, M. Tasevsky, T. Tashiro, E. Tassi, A. Tavares Delgado, Y. Tayalati, A. C. Taylor, A. J. Taylor, G. N. Taylor, P. T. E. Taylor, W. Taylor, P. Teixeira-Dias, D. Temple, H. Ten Kate, P. K. Teng, J. J. Teoh, F. Tepel, S. Terada, K. Terashi, J. Terron, S. Terzo, M. Testa, R. J. Teuscher, S. J. Thais, T. Theveneaux-Pelzer, F. Thiele, J. P. Thomas, J. Thomas-Wilsker, P. D. Thompson, A. S. Thompson, L. A. Thomsen, E. Thomson, Y. Tian, M. J. Tibbetts, R. E. Ticse Torres, V. O. Tikhomirov, Yu. A. Tikhonov, S. Timoshenko, P. Tipton, S. Tisserant, K. Todome, S. Todorova-Nova, S. Todt, J. Tojo, S. Tokár, K. Tokushuku, E. Tolley, L. Tomlinson, M. Tomoto, L. Tompkins, K. Toms, B. Tong, P. Tornambe, E. Torrence, H. Torres, E. Torró Pastor, J. Toth, F. Touchard, D. R. Tovey, C. J. Treado, T. Trefzger, F. Tresoldi, A. Tricoli, I. M. Trigger, S. Trincaz-Duvoid, M. F. Tripiana, W. Trischuk, B. Trocmé, A. Trofymov, C. Troncon, M. Trottier-McDonald, M. Trovatelli, L. Truong, M. Trzebinski, A. Trzupek, K. W. Tsang, J. C.-L. Tseng, P. V. Tsiareshka, G. Tsipolitis, N. Tsirintanis, S. Tsiskaridze, V. Tsiskaridze, E. G. Tskhadadze, I. I. Tsukerman, V. Tsulaia, S. Tsuno, D. Tsybychev, Y. Tu, A. Tudorache, V. Tudorache, T. T. Tulbure, A. N. Tuna, S. Turchikhin, D. Turgeman, I. Turk Cakir, R. Turra, P. M. Tuts, G. Ucchielli, I. Ueda, M. Ughetto, F. Ukegawa, G. Unal, A. Undrus, G. Unel, F. C. Ungaro, Y. Unno, K. Uno, C. Unverdorben, J. Urban, P. Urquijo, P. Urrejola, G. Usai, J. Usui, L. Vacavant, V. Vacek, B. Vachon, K. O. H. Vadla, A. Vaidya, C. Valderanis, E. Valdes Santurio, M. Valente, S. Valentinetti, A. Valero, L. Valéry, S. Valkar, A. Vallier, J. A. Valls Ferrer, W. Van Den Wollenberg, H. van der Graaf, P. van Gemmeren, J. Van Nieuwkoop, I. van Vulpen, M. C. van Woerden, M. Vanadia, W. Vandelli, A. Vaniachine, P. Vankov, G. Vardanyan, R. Vari, E. W. Varnes, C. Varni, T. Varol, D. Varouchas, A. Vartapetian, K. E. Varvell, J. G. Vasquez, G. A. Vasquez, F. Vazeille, D. Vazquez Furelos, T. Vazquez Schroeder, J. Veatch, V. Veeraraghavan, L. M. Veloce, F. Veloso, S. Veneziano, A. Ventura, M. Venturi, N. Venturi, A. Venturini, V. Vercesi, M. Verducci, W. Verkerke, A. T. Vermeulen, J. C. Vermeulen, M. C. Vetterli, N. Viaux Maira, O. Viazlo, I. Vichou, T. Vickey, O. E. Vickey Boeriu, G. H. A. Viehhauser, S. Viel, L. Vigani, M. Villa, M. Villaplana Perez, E. Vilucchi, M. G. Vincter, V. B. Vinogradov, A. Vishwakarma, C. Vittori, I. Vivarelli, S. Vlachos, M. Vogel, P. Vokac, G. Volpi, H. von der Schmitt, E. von Toerne, V. Vorobel, K. Vorobev, M. Vos, R. Voss, J. H. Vossebeld, N. Vranjes, M. Vranjes Milosavljevic, V. Vrba, M. Vreeswijk, R. Vuillermet, I. Vukotic, P. Wagner, W. Wagner, J. Wagner-Kuhr, H. Wahlberg, S. Wahrmund, K. Wakamiya, J. Walder, R. Walker, W. Walkowiak, V. Wallangen, C. Wang, C. Wang, F. Wang, H. Wang, H. Wang, J. Wang, J. Wang, Q. Wang, R.-J. Wang, R. Wang, S. M. Wang, T. Wang, W. Wang, W. Wang, Z. Wang, C. Wanotayaroj, A. Warburton, C. P. Ward, D. R. Wardrope, A. Washbrook, P. M. Watkins, A. T. Watson, M. F. Watson, G. Watts, S. Watts, B. M. Waugh, A. F. Webb, S. Webb, M. S. Weber, S. W. Weber, S. W. Weber, S. A. Weber, J. S. Webster, A. R. Weidberg, B. Weinert, J. Weingarten, M. Weirich, C. Weiser, H. Weits, P. S. Wells, T. Wenaus, T. Wengler, S. Wenig, N. Wermes, M. D. Werner, P. Werner, M. Wessels, T. D. Weston, K. Whalen, N. L. Whallon, A. M. Wharton, A. S. White, A. White, M. J. White, R. White, D. Whiteson, B. W. Whitmore, F. J. Wickens, W. Wiedenmann, M. Wielers, C. Wiglesworth, L. A. M. Wiik-Fuchs, A. Wildauer, F. Wilk, H. G. Wilkens, H. H. Williams, S. Williams, C. Willis, S. Willocq, J. A. Wilson, I. Wingerter-Seez, E. Winkels, F. Winklmeier, O. J. Winston, B. T. Winter, M. Wittgen, M. Wobisch, A. Wolf, T. M. H. Wolf, R. Wolff, M. W. Wolter, H. Wolters, V. W. S. Wong, N. L. Woods, S. D. Worm, B. K. Wosiek, J. Wotschack, K. W. Wozniak, M. Wu, S. L. Wu, X. Wu, Y. Wu, T. R. Wyatt, B. M. Wynne, S. Xella, Z. Xi, L. Xia, D. Xu, L. Xu, T. Xu, W. Xu, B. Yabsley, S. Yacoob, D. Yamaguchi, Y. Yamaguchi, A. Yamamoto, S. Yamamoto, T. Yamanaka, F. Yamane, M. Yamatani, T. Yamazaki, Y. Yamazaki, Z. Yan, H. Yang, H. Yang, Y. Yang, Z. Yang, W-M. Yao, Y. C. Yap, Y. Yasu, E. Yatsenko, K. H. Yau Wong, J. Ye, S. Ye, I. Yeletskikh, E. Yigitbasi, E. Yildirim, K. Yorita, K. Yoshihara, C. Young, C. J. S. Young, J. Yu, J. Yu, S. P. Y. Yuen, I. Yusuff, B. Zabinski, G. Zacharis, R. Zaidan, A. M. Zaitsev, N. Zakharchuk, J. Zalieckas, A. Zaman, S. Zambito, D. Zanzi, C. Zeitnitz, G. Zemaityte, A. Zemla, J. C. Zeng, Q. Zeng, O. Zenin, T. Ženiš, D. Zerwas, D. Zhang, D. Zhang, F. Zhang, G. Zhang, H. Zhang, J. Zhang, L. Zhang, L. Zhang, M. Zhang, P. Zhang, R. Zhang, R. Zhang, X. Zhang, Y. Zhang, Z. Zhang, X. Zhao, Y. Zhao, Z. Zhao, A. Zhemchugov, B. Zhou, C. Zhou, L. Zhou, M. Zhou, M. Zhou, N. Zhou, Y. Zhou, C. G. Zhu, H. Zhu, J. Zhu, Y. Zhu, X. Zhuang, K. Zhukov, A. Zibell, D. Zieminska, N. I. Zimine, C. Zimmermann, S. Zimmermann, Z. Zinonos, M. Zinser, M. Ziolkowski, L. Živković, G. Zobernig, A. Zoccoli, R. Zou, M. zur Nedden, L. Zwalinski, ATLAS Collaboration

**Affiliations:** 1 Department of Physics, University of Adelaide, Adelaide, Australia; 2 Physics Department, SUNY Albany, Albany, NY USA; 3 Department of Physics, University of Alberta, Edmonton, AB Canada; 4 Department of Physics, Ankara University, Ankara, Turkey; 5 Istanbul Aydin University, Istanbul, Turkey; 6 Division of Physics, TOBB University of Economics and Technology, Ankara, Turkey; 7 LAPP, CNRS/IN2P3 and Université Savoie Mont Blanc, Annecy-le-Vieux, France; 8 High Energy Physics Division, Argonne National Laboratory, Argonne, IL USA; 9 Department of Physics, University of Arizona, Tucson, AZ USA; 10 Department of Physics, The University of Texas at Arlington, Arlington, TX USA; 11 Physics Department, National and Kapodistrian University of Athens, Athens, Greece; 12 Physics Department, National Technical University of Athens, Zografou, Greece; 13 Department of Physics, The University of Texas at Austin, Austin, TX USA; 14 Institute of Physics, Azerbaijan Academy of Sciences, Baku, Azerbaijan; 15 Institut de Física d’Altes Energies (IFAE), The Barcelona Institute of Science and Technology, Barcelona, Spain; 16 Institute of Physics, University of Belgrade, Belgrade, Serbia; 17 Department for Physics and Technology, University of Bergen, Bergen, Norway; 18 Physics Division, Lawrence Berkeley National Laboratory, University of California, Berkeley, CA USA; 19 Department of Physics, Humboldt University, Berlin, Germany; 20 Albert Einstein Center for Fundamental Physics, Laboratory for High Energy Physics, University of Bern, Bern, Switzerland; 21 School of Physics and Astronomy, University of Birmingham, Birmingham, UK; 22 Department of Physics, Bogazici University, Istanbul, Turkey; 23 Department of Physics Engineering, Gaziantep University, Gaziantep, Turkey; 24 Faculty of Engineering and Natural Sciences, Istanbul Bilgi University, Istanbul, Turkey; 25 Faculty of Engineering and Natural Sciences, Bahcesehir University, Istanbul, Turkey; 26 Centro de Investigaciones, Universidad Antonio Narino, Bogotá, Colombia; 27 INFN Sezione di Bologna, Bologna, Italy; 28 Dipartimento di Fisica e Astronomia, Università di Bologna, Bologna, Italy; 29 Physikalisches Institut, University of Bonn, Bonn, Germany; 30 Department of Physics, Boston University, Boston, MA USA; 31 Department of Physics, Brandeis University, Waltham, MA USA; 32 Universidade Federal do Rio De Janeiro COPPE/EE/IF, Rio de Janeiro, Brazil; 33 Electrical Circuits Department, Federal University of Juiz de Fora (UFJF), Juiz de Fora, Brazil; 34 Federal University of Sao Joao del Rei (UFSJ), Sao Joao del Rei, Brazil; 35 Instituto de Fisica, Universidade de Sao Paulo, São Paulo, Brazil; 36 Physics Department, Brookhaven National Laboratory, Upton, NY USA; 37 Transilvania University of Brasov, Brasov, Romania; 38 Horia Hulubei National Institute of Physics and Nuclear Engineering, Bucharest, Romania; 39 Department of Physics, Alexandru Ioan Cuza University of Iasi, Iasi, Romania; 40 Physics Department, National Institute for Research and Development of Isotopic and Molecular Technologies, Cluj-Napoca, Romania; 41 University Politehnica Bucharest, Bucharest, Romania; 42 West University in Timisoara, Timisoara, Romania; 43 Departamento de Física, Universidad de Buenos Aires, Buenos Aires, Argentina; 44 Cavendish Laboratory, University of Cambridge, Cambridge, UK; 45 Department of Physics, Carleton University, Ottawa, ON Canada; 46 CERN, Geneva, Switzerland; 47 Enrico Fermi Institute, University of Chicago, Chicago, IL USA; 48 Departamento de Física, Pontificia Universidad Católica de Chile, Santiago, Chile; 49 Departamento de Física, Universidad Técnica Federico Santa María, Valparaiso, Chile; 50 Institute of High Energy Physics, Chinese Academy of Sciences, Beijing, China; 51 Department of Physics, Nanjing University, Nanjing, Jiangsu China; 52 Physics Department, Tsinghua University, Beijing, 100084 China; 53 University of Chinese Academy of Science (UCAS), Beijing, China; 54 Department of Modern Physics and State Key Laboratory of Particle Detection and Electronics, University of Science and Technology of China, Hefei, Anhui China; 55 School of Physics, Shandong University, Jinan, Shandong China; 56 Department of Physics and Astronomy, Key Laboratory for Particle Physics, Astrophysics and Cosmology, Ministry of Education, Shanghai Key Laboratory for Particle Physics and Cosmology, Shanghai Jiao Tong University, Shanghai (also at PKU-CHEP), Shanghai, China; 57 Université Clermont Auvergne, CNRS/IN2P3, LPC, Clermont-Ferrand, France; 58 Nevis Laboratory, Columbia University, Irvington, NY USA; 59 Niels Bohr Institute, University of Copenhagen, Copenhagen, Denmark; 60 INFN Gruppo Collegato di Cosenza, Laboratori Nazionali di Frascati, Frascati, Italy; 61 Dipartimento di Fisica, Università della Calabria, Rende, Italy; 62 Faculty of Physics and Applied Computer Science, AGH University of Science and Technology, Kraków, Poland; 63 Marian Smoluchowski Institute of Physics, Jagiellonian University, Kraków, Poland; 64 Institute of Nuclear Physics, Polish Academy of Sciences, Kraków, Poland; 65 Physics Department, Southern Methodist University, Dallas, TX USA; 66 Physics Department, University of Texas at Dallas, Richardson, TX USA; 67 DESY, Hamburg and Zeuthen, Germany; 68 Lehrstuhl für Experimentelle Physik IV, Technische Universität Dortmund, Dortmund, Germany; 69 Institut für Kern- und Teilchenphysik, Technische Universität Dresden, Dresden, Germany; 70 Department of Physics, Duke University, Durham, NC USA; 71 SUPA-School of Physics and Astronomy, University of Edinburgh, Edinburgh, UK; 72 INFN Laboratori Nazionali di Frascati, Frascati, Italy; 73 Fakultät für Mathematik und Physik, Albert-Ludwigs-Universität, Freiburg, Germany; 74 Departement de Physique Nucleaire et Corpusculaire, Université de Genève, Geneva, Switzerland; 75 INFN Sezione di Genova, Genoa, Italy; 76 Dipartimento di Fisica, Università di Genova, Genoa, Italy; 77 E. Andronikashvili Institute of Physics, Iv. Javakhishvili Tbilisi State University, Tbilisi, Georgia; 78 High Energy Physics Institute, Tbilisi State University, Tbilisi, Georgia; 79 II Physikalisches Institut, Justus-Liebig-Universität Giessen, Giessen, Germany; 80 SUPA-School of Physics and Astronomy, University of Glasgow, Glasgow, UK; 81 II Physikalisches Institut, Georg-August-Universität, Göttingen, Germany; 82 Laboratoire de Physique Subatomique et de Cosmologie, Université Grenoble-Alpes, CNRS/IN2P3, Grenoble, France; 83 Laboratory for Particle Physics and Cosmology, Harvard University, Cambridge, MA USA; 84 Kirchhoff-Institut für Physik, Ruprecht-Karls-Universität Heidelberg, Heidelberg, Germany; 85 Physikalisches Institut, Ruprecht-Karls-Universität Heidelberg, Heidelberg, Germany; 86 Faculty of Applied Information Science, Hiroshima Institute of Technology, Hiroshima, Japan; 87 Department of Physics, The Chinese University of Hong Kong, Shatin, NT Hong Kong; 88 Department of Physics, The University of Hong Kong, Hong Kong, China; 89 Department of Physics, Institute for Advanced Study, The Hong Kong University of Science and Technology, Clear Water Bay, Kowloon, Hong Kong, China; 90 Department of Physics, National Tsing Hua University, Taiwan, Taiwan; 91 Department of Physics, Indiana University, Bloomington, IN USA; 92 Institut für Astro- und Teilchenphysik, Leopold-Franzens-Universität, Innsbruck, Austria; 93 University of Iowa, Iowa City, IA USA; 94 Department of Physics and Astronomy, Iowa State University, Ames, IA USA; 95 Joint Institute for Nuclear Research, JINR Dubna, Dubna, Russia; 96 KEK, High Energy Accelerator Research Organization, Tsukuba, Japan; 97 Graduate School of Science, Kobe University, Kobe, Japan; 98 Faculty of Science, Kyoto University, Kyoto, Japan; 99 Kyoto University of Education, Kyoto, Japan; 100 Research Center for Advanced Particle Physics and Department of Physics, Kyushu University, Fukuoka, Japan; 101 Instituto de Física La Plata, Universidad Nacional de La Plata and CONICET, La Plata, Argentina; 102 Physics Department, Lancaster University, Lancaster, UK; 103 INFN Sezione di Lecce, Lecce, Italy; 104 Dipartimento di Matematica e Fisica, Università del Salento, Lecce, Italy; 105 Oliver Lodge Laboratory, University of Liverpool, Liverpool, UK; 106 Department of Experimental Particle Physics, Jožef Stefan Institute and Department of Physics, University of Ljubljana, Ljubljana, Slovenia; 107 School of Physics and Astronomy, Queen Mary University of London, London, UK; 108 Department of Physics, Royal Holloway University of London, Surrey, UK; 109 Department of Physics and Astronomy, University College London, London, UK; 110 Louisiana Tech University, Ruston, LA USA; 111 Laboratoire de Physique Nucléaire et de Hautes Energies, UPMC and Université Paris-Diderot and CNRS/IN2P3, Paris, France; 112 Fysiska institutionen, Lunds universitet, Lund, Sweden; 113 Departamento de Fisica Teorica C-15, Universidad Autonoma de Madrid, Madrid, Spain; 114 Institut für Physik, Universität Mainz, Mainz, Germany; 115 School of Physics and Astronomy, University of Manchester, Manchester, UK; 116 CPPM, Aix-Marseille Université and CNRS/IN2P3, Marseille, France; 117 Department of Physics, University of Massachusetts, Amherst, MA USA; 118 Department of Physics, McGill University, Montreal, QC Canada; 119 School of Physics, University of Melbourne, Victoria, Australia; 120 Department of Physics, The University of Michigan, Ann Arbor, MI USA; 121 Department of Physics and Astronomy, Michigan State University, East Lansing, MI USA; 122 INFN Sezione di Milano, Milan, Italy; 123 Dipartimento di Fisica, Università di Milano, Milan, Italy; 124 B.I. Stepanov Institute of Physics, National Academy of Sciences of Belarus, Minsk, Republic of Belarus; 125 Research Institute for Nuclear Problems of Byelorussian State University, Minsk, Republic of Belarus; 126 Group of Particle Physics, University of Montreal, Montreal, QC Canada; 127 P.N. Lebedev Physical Institute of the Russian Academy of Sciences, Moscow, Russia; 128 Institute for Theoretical and Experimental Physics (ITEP), Moscow, Russia; 129 National Research Nuclear University MEPhI, Moscow, Russia; 130 D.V. Skobeltsyn Institute of Nuclear Physics, M.V. Lomonosov Moscow State University, Moscow, Russia; 131 Fakultät für Physik, Ludwig-Maximilians-Universität München, Munich, Germany; 132 Max-Planck-Institut für Physik (Werner-Heisenberg-Institut), Munich, Germany; 133 Nagasaki Institute of Applied Science, Nagasaki, Japan; 134 Graduate School of Science and Kobayashi-Maskawa Institute, Nagoya University, Nagoya, Japan; 135 INFN Sezione di Napoli, Naples, Italy; 136 Dipartimento di Fisica, Università di Napoli, Naples, Italy; 137 Department of Physics and Astronomy, University of New Mexico, Albuquerque, NM USA; 138 Institute for Mathematics, Astrophysics and Particle Physics, Radboud University Nijmegen/Nikhef, Nijmegen, The Netherlands; 139 Nikhef National Institute for Subatomic Physics, University of Amsterdam, Amsterdam, The Netherlands; 140 Department of Physics, Northern Illinois University, DeKalb, IL USA; 141 Budker Institute of Nuclear Physics, SB RAS, Novosibirsk, Russia; 142 Department of Physics, New York University, New York, NY USA; 143 Ohio State University, Columbus, OH USA; 144 Faculty of Science, Okayama University, Okayama, Japan; 145 Homer L. Dodge Department of Physics and Astronomy, University of Oklahoma, Norman, OK USA; 146 Department of Physics, Oklahoma State University, Stillwater, OK USA; 147 Palacký University, RCPTM, Olomouc, Czech Republic; 148 Center for High Energy Physics, University of Oregon, Eugene, OR USA; 149 LAL, Univ. Paris-Sud, CNRS/IN2P3, Université Paris-Saclay, Orsay, France; 150 Graduate School of Science, Osaka University, Osaka, Japan; 151 Department of Physics, University of Oslo, Oslo, Norway; 152 Department of Physics, Oxford University, Oxford, UK; 153 INFN Sezione di Pavia, Pavia, Italy; 154 Dipartimento di Fisica, Università di Pavia, Pavia, Italy; 155 Department of Physics, University of Pennsylvania, Philadelphia, PA USA; 156 National Research Centre “Kurchatov Institute” B.P. Konstantinov Petersburg Nuclear Physics Institute, St. Petersburg, Russia; 157 INFN Sezione di Pisa, Pisa, Italy; 158 Dipartimento di Fisica E. Fermi, Università di Pisa, Pisa, Italy; 159 Department of Physics and Astronomy, University of Pittsburgh, Pittsburgh, PA USA; 160 Laboratório de Instrumentação e Física Experimental de Partículas-LIP, Lisbon, Portugal; 161 Faculdade de Ciências, Universidade de Lisboa, Lisbon, Portugal; 162 Department of Physics, University of Coimbra, Coimbra, Portugal; 163 Centro de Física Nuclear da Universidade de Lisboa, Lisbon, Portugal; 164 Departamento de Fisica, Universidade do Minho, Braga, Portugal; 165 Departamento de Fisica Teorica y del Cosmos, Universidad de Granada, Granada, Spain; 166 Dep Fisica and CEFITEC of Faculdade de Ciencias e Tecnologia, Universidade Nova de Lisboa, Caparica, Portugal; 167 Institute of Physics, Academy of Sciences of the Czech Republic, Prague, Czech Republic; 168 Czech Technical University in Prague, Prague, Czech Republic; 169 Faculty of Mathematics and Physics, Charles University, Prague, Czech Republic; 170 State Research Center Institute for High Energy Physics (Protvino), NRC KI, Protvino, Russia; 171 Particle Physics Department, Rutherford Appleton Laboratory, Didcot, UK; 172 INFN Sezione di Roma, Rome, Italy; 173 Dipartimento di Fisica, Sapienza Università di Roma, Rome, Italy; 174 INFN Sezione di Roma Tor Vergata, Rome, Italy; 175 Dipartimento di Fisica, Università di Roma Tor Vergata, Rome, Italy; 176 INFN Sezione di Roma Tre, Rome, Italy; 177 Dipartimento di Matematica e Fisica, Università Roma Tre, Rome, Italy; 178 Faculté des Sciences Ain Chock, Réseau Universitaire de Physique des Hautes Energies-Université Hassan II, Casablanca, Morocco; 179 Centre National de l’Energie des Sciences Techniques Nucleaires, Rabat, Morocco; 180 Faculté des Sciences Semlalia, Université Cadi Ayyad, LPHEA-Marrakech, Marrakech, Morocco; 181 Faculté des Sciences, Université Mohamed Premier and LPTPM, Oujda, Morocco; 182 Faculté des Sciences, Université Mohammed V, Rabat, Morocco; 183 DSM/IRFU (Institut de Recherches sur les Lois Fondamentales de l’Univers), CEA Saclay (Commissariat à l’Energie Atomique et aux Energies Alternatives), Gif-sur-Yvette, France; 184 Santa Cruz Institute for Particle Physics, University of California Santa Cruz, Santa Cruz, CA USA; 185 Department of Physics, University of Washington, Seattle, WA USA; 186 Department of Physics and Astronomy, University of Sheffield, Sheffield, UK; 187 Department of Physics, Shinshu University, Nagano, Japan; 188 Department Physik, Universität Siegen, Siegen, Germany; 189 Department of Physics, Simon Fraser University, Burnaby, BC Canada; 190 SLAC National Accelerator Laboratory, Stanford, CA USA; 191 Faculty of Mathematics, Physics and Informatics, Comenius University, Bratislava, Slovak Republic; 192 Department of Subnuclear Physics, Institute of Experimental Physics of the Slovak Academy of Sciences, Kosice, Slovak Republic; 193 Department of Physics, University of Cape Town, Cape Town, South Africa; 194 Department of Physics, University of Johannesburg, Johannesburg, South Africa; 195 School of Physics, University of the Witwatersrand, Johannesburg, South Africa; 196 Department of Physics, Stockholm University, Stockholm, Sweden; 197 The Oskar Klein Centre, Stockholm, Sweden; 198 Physics Department, Royal Institute of Technology, Stockholm, Sweden; 199 Departments of Physics and Astronomy and Chemistry, Stony Brook University, Stony Brook, NY USA; 200 Department of Physics and Astronomy, University of Sussex, Brighton, UK; 201 School of Physics, University of Sydney, Sydney, Australia; 202 Institute of Physics, Academia Sinica, Taipei, Taiwan; 203 Department of Physics, Technion: Israel Institute of Technology, Haifa, Israel; 204 Raymond and Beverly Sackler School of Physics and Astronomy, Tel Aviv University, Tel Aviv, Israel; 205 Department of Physics, Aristotle University of Thessaloniki, Thessaloníki, Greece; 206 International Center for Elementary Particle Physics and Department of Physics, The University of Tokyo, Tokyo, Japan; 207 Graduate School of Science and Technology, Tokyo Metropolitan University, Tokyo, Japan; 208 Department of Physics, Tokyo Institute of Technology, Tokyo, Japan; 209 Tomsk State University, Tomsk, Russia; 210 Department of Physics, University of Toronto, Toronto, ON Canada; 211 INFN-TIFPA, Trento, Italy; 212 University of Trento, Trento, Italy; 213 TRIUMF, Vancouver, BC Canada; 214 Department of Physics and Astronomy, York University, Toronto, ON Canada; 215 Faculty of Pure and Applied Sciences, and Center for Integrated Research in Fundamental Science and Engineering, University of Tsukuba, Tsukuba, Japan; 216 Department of Physics and Astronomy, Tufts University, Medford, MA USA; 217 Department of Physics and Astronomy, University of California Irvine, Irvine, CA USA; 218 INFN Gruppo Collegato di Udine, Sezione di Trieste, Udine, Italy; 219 ICTP, Trieste, Italy; 220 Dipartimento di Chimica, Fisica e Ambiente, Università di Udine, Udine, Italy; 221 Department of Physics and Astronomy, University of Uppsala, Uppsala, Sweden; 222 Department of Physics, University of Illinois, Urbana, IL USA; 223 Instituto de Fisica Corpuscular (IFIC), Centro Mixto Universidad de Valencia - CSIC, Valencia, Spain; 224 Department of Physics, University of British Columbia, Vancouver, BC Canada; 225 Department of Physics and Astronomy, University of Victoria, Victoria, BC Canada; 226 Department of Physics, University of Warwick, Coventry, UK; 227 Waseda University, Tokyo, Japan; 228 Department of Particle Physics, The Weizmann Institute of Science, Rehovot, Israel; 229 Department of Physics, University of Wisconsin, Madison, WI USA; 230 Fakultät für Physik und Astronomie, Julius-Maximilians-Universität, Würzburg, Germany; 231 Fakultät für Mathematik und Naturwissenschaften, Fachgruppe Physik, Bergische Universität Wuppertal, Wuppertal, Germany; 232 Department of Physics, Yale University, New Haven, CT USA; 233 Yerevan Physics Institute, Yerevan, Armenia; 234 Centre de Calcul de l’Institut National de Physique Nucléaire et de Physique des Particules (IN2P3), Villeurbanne, France; 235 Academia Sinica Grid Computing, Institute of Physics, Academia Sinica, Taipei, Taiwan; 236 CERN, 1211 Geneva 23, Switzerland

## Abstract

Measurements of longitudinal flow correlations are presented for charged particles in the pseudorapidity range $|\eta |<2.4$ using 7 and 470 $\upmu \hbox {b}^{-1}$ of Pb+Pb collisions at $\sqrt{s_{\text {NN}}}=2.76$ and 5.02 TeV, respectively, recorded by the ATLAS detector at the LHC. It is found that the correlation between the harmonic flow coefficients $v_n$ measured in two separated $\eta$ intervals does not factorise into the product of single-particle coefficients, and this breaking of factorisation, or flow decorrelation, increases linearly with the $\eta$ separation between the intervals. The flow decorrelation is stronger at 2.76 TeV than at 5.02 TeV. Higher-order moments of the correlations are also measured, and the corresponding linear coefficients for the $k{\text {th}}$-moment of the $v_n$ are found to be proportional to *k* for $v_3$, but not for $v_2$. The decorrelation effect is separated into contributions from the magnitude of $v_n$ and the event-plane orientation, each as a function of $\eta$. These two contributions are found to be comparable. The longitudinal flow correlations are also measured between $v_n$ of different order in *n*. The decorrelations of $v_2$ and $v_3$ are found to be independent of each other, while the decorrelations of $v_4$ and $v_5$ are found to be driven by the nonlinear contribution from $v_2^2$ and $v_2v_3$, respectively.

## Introduction

Heavy-ion collisions at RHIC and the LHC create hot, dense matter whose space-time evolution is well described by relativistic viscous hydrodynamics [1, 2]. Owing to strong event-by-event (EbyE) density fluctuations in the initial state, the space-time evolution of the produced matter also fluctuates event by event. These fluctuations lead to correlations of particle multiplicity in momentum space in both the transverse and longitudinal directions with respect to the collision axis. Studies of particle correlations in the transverse plane have revealed strong harmonic modulation of the particle densities in the azimuthal angle: d$N/{\text {d}}\phi \propto 1+2\sum _{n=1}^{\infty }v_{n}\cos n(\phi -\Phi _{n})$, where $v_n$ and $\Phi _n$ represent the magnitude and event-plane angle of the $n^{\mathrm {th}}$-order harmonic flow. The measurements of harmonic flow coefficients $v_n$ and their EbyE fluctuations, as well as the correlations between $\Phi _{n}$ of different order [3–9], have placed important constraints on the properties of the dense matter and on transverse density fluctuations in the initial state [10–15].

Most previous flow studies assumed that the initial condition and space-time evolution of the matter are boost-invariant in the longitudinal direction. Recent model studies of two-particle correlations as a function of pseudorapidity $\eta$ revealed strong EbyE fluctuations of the flow magnitude and phase between two well-separated pseudorapidities, i.e. $v_n(\eta _1)\ne v_n(\eta _2)$ (forward-backward or FB asymmetry) and $\Phi _{n}(\eta _1)\ne \Phi _{n}(\eta _2)$ (event-plane twist) [16–18]. The CMS Collaboration proposed an observable based on the ratio of two correlations: the correlation between $\eta$ and $\eta _{\mathrm {ref}}$ and the correlation between $-\eta$ and $\eta _{\mathrm {ref}}$. This ratio is sensitive to the correlation between $\eta$ and $-\eta$ [19]. The CMS results show that the longitudinal fluctuations lead to a linear decrease of the ratio with $\eta$, and the slope of the decrease shows a strong centrality dependence for elliptic flow $v_2$ but very weak dependences for $v_3$ and $v_4$. This paper extends the CMS result by measuring several new observables based on multi-particle correlations in two or more $\eta$ intervals [20]. These observables are sensitive to the EbyE fluctuations of the initial condition in the longitudinal direction. They are also sensitive to nonlinear mode-mixing effects, e.g. $v_4$ contains nonlinear contributions that are proportional to $v_2^2$ [8, 9, 21–23]. Furthermore, the measurements are performed at two nucleon–nucleon centre-of-mass collision energies, $\sqrt{s_{\text {NN}}}=2.76$ TeV and 5.02 TeV, to evaluate the $\sqrt{s_{\text {NN}}}$ dependence of the longitudinal flow fluctuations. Recent model calculations predict an increase of longitudinal flow fluctuations at lower $\sqrt{s_{\text {NN}}}$ [24]. Therefore, measurements of these observables at two collision energies can provide new insights into the initial condition along the longitudinal direction and should help in the development of full three-dimensional viscous hydrodynamic models.

Using these new observables, this paper improves the study of the longitudinal dynamics of collective flow in three ways. Firstly, the CMS measurement, which is effectively the first moment of the correlation between $v_n$ in separate $\eta$ intervals, is extended to the second and the third moments. Secondly, a correlation between four different $\eta$ intervals is measured to estimate the contributions from the fluctuations of $v_n$ amplitudes as well as the contributions from fluctuations of $\Phi _{n}$. Thirdly, correlations between harmonics of different order are also measured, e.g. between $v_2$ and $v_4$ in different $\eta$ intervals, to investigate how mode-mixing effects evolve with rapidity. In this way, this paper presents a measurement of flow decorrelation involving $v_2$, $v_3$, $v_4$ and $v_5$, using Pb+Pb collisions at $\sqrt{s_{\text {NN}}}=2.76$ and 5.02 TeV.

## Observables

This section gives a brief summary of the observables measured in this paper, further details can be found in Refs. [19, 20, 25]. The azimuthal anisotropy of the particle production in an event is conveniently described by harmonic flow vectors ${\varvec{V}}_n = v_n e^{{\text {i}}n\Phi _n}$ 1, where $v_n$ and $\Phi _n$ are the magnitude and phase (or event plane), respectively. The ${\varvec{V}}_n$ are estimated from the observed per-particle normalised flow vector ${\varvec{q}}_n$ [5]:

1 $$\begin{aligned} {\varvec{q}}_{n} \equiv \frac{{\sum _{i}} {w_{i}}e^{{\text{i}}n{\phi_{i}}}}{{\sum _i}{w_i}}. \end{aligned}$$

The sums run over all particles in a given $\eta$ interval of the event, and $\phi _i$ and $w_i$ are the azimuthal angle and the weight assigned to the $i{\text {th}}$ particle, respectively. The weight accounts for detector non-uniformity and tracking inefficiency.

**Fig. 1:**
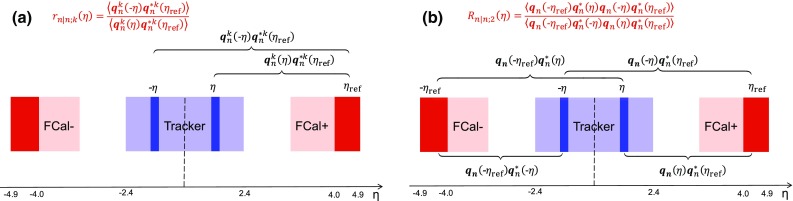
Schematic illustration of the procedure for constructing the corrlators $r_{n|n;k}(\eta )$ Eq. (2) (left panel) and $R_{n|n;2}(\eta )$ Eq. (5) (right panel). The acceptance coverages for the ATLAS tracker used for $\eta$ and reference detector used for $\eta _{\mathrm {ref}}$ are discussed in Sect. 5

The longitudinal flow fluctuations are studied using the correlation between the $k{\text {th}}$-moment of the $n{\text {th}}$-order flow vectors in two different $\eta$ intervals, averaged over events in a given centrality interval, $r_{n|n;k}$, for $k=1$,2,3:

2 $$\begin{aligned} r_{n|n;k}(\eta )= & {} \frac{\left\langle {\varvec{q}}_n^k (-\eta ) {\varvec{q}}_n^{*k}(\eta _{\mathrm {ref}})\right\rangle }{\left\langle {\varvec{q}}_n^k (\eta ){\varvec{q}}_n^{*k}(\eta _{\mathrm {ref}})\right\rangle }\nonumber \\= & {} \frac{\left\langle \left[ v_n(-\eta ) v_n(\eta _{\mathrm {ref}})\right] ^k \cos kn(\Phi _n(-\eta )-\Phi _n(\eta _{\mathrm {ref}}))\right\rangle }{\left\langle \left[ v_n(\eta ) v_n(\eta _{\mathrm {ref}})\right] ^k \cos kn(\Phi _n(\eta )-\Phi _n(\eta _{\mathrm {ref}}))\right\rangle }\;, \end{aligned}$$

where $\eta _{\mathrm {ref}}$ is the reference pseudorapidity common to the numerator and the denominator, the subscript “*n*|*n*; *k*” denotes the $k{\text {th}}$-moment of the flow vectors of order *n* at $\eta$, combined with the $k{\text {th}}$ moment of the conjugate of the flow vector of order *n* at $\eta _{\mathrm {ref}}$. The sine terms vanish in the last expression in Eq. (2) because any observable must be an even function of $\Phi _n(-\eta )-\Phi _n(\eta _{\mathrm {ref}})$. A schematic illustration of the choice of the $\eta$ ($|\eta |<2.4$) and $\eta _{\mathrm {ref}}$ ($4.0<|\eta _{\mathrm {ref}}|<4.9$) to be discussed in Sect. 5, as well as the relations between different flow vectors, are shown in the left panel of Fig. 1. This observable is effectively a 2k-particle correlator between two subevents as defined in Ref. [28], and the particle multiplets containing duplicated particle indices are removed using the cumulant framework, with particle weights taken into account [20].

The observable measured by the CMS Collaboration [19] corresponds to $k=1$, i.e. $r_{n|n;1}$ . It should be noted that $\left\langle {\varvec{q}}_n\right\rangle =0$ because the event plane changes randomly from event to event. Hence a direct study of the correlation between $+\eta$ and $-\eta$ via a quantity such as $\left\langle {\varvec{q}}_n (+\eta ) {\varvec{q}}_n^{*}(-\eta )\right\rangle /(\left\langle {\varvec{q}}_n (+\eta )\right\rangle \left\langle {\varvec{q}}_n^{*} (-\eta )\right\rangle )$ is not possible. One could also consider a quantity like $\left\langle {\varvec{q}}_n (+\eta ) {\varvec{q}}_n^{*}(-\eta )\right\rangle /\left( \left\langle q_n^2 (\eta )\right\rangle \left\langle q_n^2 (-\eta )\right\rangle \right) ^{1/2}$, but the denominator would be affected by short-range correlations. Hence, it is preferable to work with quantities of the type used in Eq. (2), which give a correlator sensitive to the flow decorrelation between $\eta$ and $-\eta$ through the reference flow vector ${\varvec{q}}_n^{k}(\eta _{\mathrm {ref}})$.

One important feature of Eq. (2) is that the detector effects at $\eta _{\mathrm {ref}}$ are expected to cancel out to a great extent (see Sect. 5). To ensure a sizeable pseudorapidity gap between the flow vectors in both the numerator and denominator of Eq. (2), $\eta _{\mathrm {ref}}$ is usually chosen to be at large pseudorapidity, e.g. $\eta _{\mathrm {ref}}>4$ or $\eta _{\mathrm {ref}}<-4$, while the pseudorapidity of ${\varvec{q}}_n(-\eta )$ and ${\varvec{q}}_n(\eta )$ is usually chosen to be close to mid-rapidity, $|\eta |<2.4$. If flow harmonics from multi-particle correlations factorise into single-particle flow harmonics, e.g. $\left\langle {\varvec{V}}_n^k (\eta ) {\varvec{V}}_n^{*k}(\eta _{\mathrm {ref}})\right\rangle ^2=\left\langle v_n^{2k} (\eta )\right\rangle \left\langle v_n^{2k}(\eta _{\mathrm {ref}})\right\rangle$, then it is expected that $r_{n|n;k}(\eta )=1$. Therefore, a value of $r_{n|n;k}(\eta )$ different from 1 implies a factorisation-breaking effect due to longitudinal flow fluctuations, and such an effect is generally referred to as “flow decorrelation”.

Based on the CMS measurement [19] and arguments in Ref. [20], the observable $r_{n|n;k}(\eta )$ is expected to be approximately a linear function of $\eta$ with a negative slope, and is sensitive to both the asymmetry in the magnitude of $v_n$ and the twist of the event-plane angles between $\eta$ and $-\eta$:

3 $$\begin{aligned} r_{n|n;k}(\eta ) \approx 1-2F_{n;k}^{\text {r}}\eta ,\;\; F_{n;k}^{\text {r}}= F_{n;k}^{\text {asy}}+F_{n;k}^{\text {twi}}, \end{aligned}$$

where $F_{n;k}^{\text {asy}}$ and $F_{n;k}^{\text {twi}}$ represent the contribution from FB $v_n$ asymmetry and event-plane twist, respectively. The $r_{n|n;k}$ results obtained in Ref. [19] were for $k=1$ and $n=2$, 3, 4. The measured $F_{n;1}^{\text {r}}$ show only a weak dependence on $\eta _{\mathrm {ref}}$ for $\eta _{\mathrm {ref}}>3$ or $\eta _{\mathrm {ref}}<-3$ at the LHC. Measuring $r_{n|n;k}$ for $k>1$ provides new information on how the $v_n$ asymmetry and event-plane twist fluctuate event by event.

If the amount of decorrelation for the $k{\text {th}}$-moment of the flow vector is proportional to *k*, it can be shown that [20]:

4 $$\begin{aligned} r_{n|n;k}\approx r_{n|n;1}^k,\;\;F_{n;k}^{\text {r}}\approx k F_{n;1}^{\text {r}}. \end{aligned}$$

Deviations from Eq. (4) are sensitive to the detailed EbyE structure of the flow fluctuations in the longitudinal direction.

To estimate the separate contributions of the asymmetry and twist effects, a new observable involving correlations of flow vectors in four $\eta$ intervals is used [20]:

5 $$\begin{aligned} R_{n|n;2}(\eta )= & {} \frac{\left\langle {\varvec{q}}_n(-\eta _{\mathrm {ref}}){\varvec{q}}_n^{*} (\eta ) {\varvec{q}}_n (-\eta ) {\varvec{q}}_n^*(\eta _{\mathrm {ref}})\right\rangle }{\left\langle {\varvec{q}}_n(-\eta _{\mathrm {ref}}){\varvec{q}}_n^* (-\eta ){\varvec{q}}_n (\eta ) {\varvec{q}}_n^*(\eta _{\mathrm {ref}})\right\rangle }\nonumber \\= & {} \frac{\left\langle v_n(-\eta _{\mathrm {ref}})v_n(-\eta )v_n(\eta )v_n(\eta _{\mathrm {ref}})\cos n\left[ \Phi _n(-\eta _{\mathrm {ref}})-\Phi _n(\eta _{\mathrm {ref}})+(\Phi _n(-\eta )-\Phi _n(\eta ))\right] \right\rangle }{\left\langle v_n(-\eta _{\mathrm {ref}})v_n(-\eta )v_n(\eta )v_n(\eta _{\mathrm {ref}})\cos n\left[ \Phi _n(-\eta _{\mathrm {ref}})-\Phi _n(\eta _{\mathrm {ref}})-(\Phi _n(-\eta )-\Phi _n(\eta ))\right] \right\rangle }, \end{aligned}$$

where the notation “2” in the subscript indicates that there are two ${\varvec{q}}_n$ and two ${\varvec{q}}_n^{*}$ in the numerator and denominator. A schematic illustration of the relations between different flow vectors is shown in the right panel of Fig. 1. Since the effect of an asymmetry is the same in both the numerator and the denominator, this correlator is mainly sensitive to the event-plane twist effects:

6 $$\begin{aligned} R_{n|n;2} (\eta ) \approx 1-2F_{n;2}^{\text {R}}\eta , F_{n;2}^{\text {R}}= F_{n;2}^{\text {twi}}. \end{aligned}$$

Therefore, the asymmetry and twist contributions can be estimated by combining Eqs. (3) and (6).

Measurements of longitudinal flow fluctuations can also be extended to correlations between harmonics of different order:

7 $$\begin{aligned} r_{2,3|2,3}(\eta )= & {} \frac{\left\langle {\varvec{q}}_2 (-\eta ){\varvec{q}}_2^{*}(\eta _{\mathrm {ref}}){\varvec{q}}_3 (-\eta ){\varvec{q}}_3^{*}(\eta _{\mathrm {ref}})\right\rangle }{\left\langle {\varvec{q}}_2 (\eta ){\varvec{q}}_2^{*}(\eta _{\mathrm {ref}}){\varvec{q}}_3 (\eta ){\varvec{q}}_3^{*}(\eta _{\mathrm {ref}})\right\rangle }, \end{aligned}$$

8 $$\begin{aligned} r_{2,2|4}(\eta )= & {} \frac{\left\langle {\varvec{q}}_2^2 (-\eta ) {\varvec{q}}_4^{*}(\eta _{\mathrm {ref}})\right\rangle +\left\langle {\varvec{q}}_2^2 (\eta _{\mathrm {ref}}) {\varvec{q}}_4^{*}(-\eta )\right\rangle }{\left\langle {\varvec{q}}_2^2 (\eta ){\varvec{q}}_4^{*}(\eta _{\mathrm {ref}})\right\rangle +\left\langle {\varvec{q}}_2^2 (\eta _{\mathrm {ref}}){\varvec{q}}_4^{*}(\eta )\right\rangle }, \end{aligned}$$

9 $$\begin{aligned} r_{2,3|5}(\eta )= & {} \frac{\left\langle {\varvec{q}}_2(-\eta ){\varvec{q}}_3(-\eta ) {\varvec{q}}_5^{*}(\eta _{\mathrm {ref}}) \right\rangle +\left\langle {\varvec{q}}_2(\eta _{\mathrm {ref}}){\varvec{q}}_3(\eta _{\mathrm {ref}}) {\varvec{q}}_5^{*}(-\eta ) \right\rangle }{\left\langle {\varvec{q}}_2 (\eta ){\varvec{q}}_3 (\eta ){\varvec{q}}_5^{*}(\eta _{\mathrm {ref}})\right\rangle +\left\langle {\varvec{q}}_2 (\eta _{\mathrm {ref}}){\varvec{q}}_3 (\eta _{\mathrm {ref}}){\varvec{q}}_5^{*}(\eta )\right\rangle }, \end{aligned}$$

where the comma in the subscripts denotes the combination of ${\varvec{q}}_n$ of different order. If the longitudinal fluctuations for ${\varvec{V}}_2$ and ${\varvec{V}}_3$ are independent of each other, one would expect $r_{2,3|2,3}= r_{2|2;1}r_{3|3;1}$ [20]. On the other hand, $r_{2,2|4}$ and $r_{2,3|5}$ are sensitive to the $\eta$ dependence of the correlations between $v_n$ and event planes of different order, for example $\left\langle {\varvec{q}}_2^2 (-\eta ) {\varvec{q}}_4^{*}(\eta _{\mathrm {ref}})\right\rangle = \left\langle v_2^2(-\eta ) v_4(\eta _{\mathrm {ref}}) \cos 4(\Phi _2(-\eta )-\Phi _4(\eta _{\mathrm {ref}}))\right\rangle$. Correlations between different orders have been measured previously at the LHC [8, 9, 23, 29].

It is well established that the ${\varvec{V}}_4$ and ${\varvec{V}}_5$ in Pb+Pb collisions contain a linear contribution associated with initial geometry and mode-mixing contributions from lower-order harmonics due to nonlinear hydrodynamic response [8, 9, 14, 21, 22]:

10 $$\begin{aligned} {\varvec{V}}_4 = {\varvec{V}}_{4\text {L}} +\chi _{4} {\varvec{V}}_2^2,\;\;{\varvec{V}}_5 = {\varvec{V}}_{5\text {L}}+\chi _{5} {\varvec{V}}_2{\varvec{V}}_3, \end{aligned}$$

where the linear component ${\varvec{V}}_{n\text {L}}$ is driven by the corresponding eccentricity in the initial geometry [11]. If the linear component of $v_4$ and $v_5$ is uncorrelated with lower-order harmonics, i.e. ${\varvec{V}}_2^2{\varvec{V}}_{4\text {L}}^{*}\sim 0$ and ${\varvec{V}}_2{\varvec{V}}_3{\varvec{V}}_{5\text {L}}^{*}\sim 0$, one expects [20]:

11 $$\begin{aligned} r_{2,2|4}\approx r_{2|2;2},\;\;r_{2,3|5}\approx r_{2,3|2,3}. \end{aligned}$$

Furthermore, using Eq. (10) the $r_{n|n;1}$ correlators involving $v_4$ and $v_5$ can be approximated by:

12 $$\begin{aligned} r_{4|4;1}(\eta )\approx & {} \frac{\left\langle {\varvec{V}}_{4\text {L}}(-\eta ){\varvec{V}}_{4\text {L}}^{*}(\eta _{\mathrm {ref}})\right\rangle +\chi _{4}^2\left\langle {\varvec{V}}_2^2 (-\eta ) {\varvec{V}}_2^{*2}(\eta _{\mathrm {ref}})\right\rangle }{\left\langle {\varvec{V}}_{4\text {L}}(\eta ){\varvec{V}}_{4\text {L}}^{*}(\eta _{\mathrm {ref}})\right\rangle +\chi _{4}^2\left\langle {\varvec{V}}_2^2 (\eta ) {\varvec{V}}_2^{*2}(\eta _{\mathrm {ref}})\right\rangle }, \end{aligned}$$

13 $$\begin{aligned} r_{5|5;1}(\eta )\approx & {} \frac{\left\langle {\varvec{V}}_{5\text {L}}(-\eta ){\varvec{V}}_{5\text {L}}^{*}(\eta _{\mathrm {ref}})\right\rangle +\chi _{5}^2\left\langle {\varvec{V}}_2 (-\eta ){\varvec{V}}_2^{*}(\eta _{\mathrm {ref}}){\varvec{V}}_3 (-\eta ){\varvec{V}}_3^{*}(\eta _{\mathrm {ref}})\right\rangle }{\left\langle {\varvec{V}}_{5\text {L}}(\eta ){\varvec{V}}_{5\text {L}}^{*}(\eta _{\mathrm {ref}})\right\rangle +\chi _{5}^2\left\langle {\varvec{V}}_2 (\eta ){\varvec{V}}_2^{*}(\eta _{\mathrm {ref}}){\varvec{V}}_3 (\eta ){\varvec{V}}_3^{*}(\eta _{\mathrm {ref}})\right\rangle }. \end{aligned}$$

Therefore, both the linear and nonlinear components are important for $r_{4|4;1}$ and $r_{5|5;1}$.

## ATLAS detector and trigger

The ATLAS detector [30] provides nearly full solid-angle coverage of the collision point with tracking detectors, calorimeters, and muon chambers, and is well suited for measurements of multi-particle correlations over a large pseudorapidity range.^2^ The measurements were performed using the inner detector (ID), minimum-bias trigger scintillators (MBTS), the forward calorimeters (FCal), and the zero-degree calorimeters (ZDC). The ID detects charged particles within $|\eta | < 2.5$ using a combination of silicon pixel detectors, silicon microstrip detectors (SCT), and a straw-tube transition-radiation tracker (TRT), all immersed in a 2 T axial magnetic field [31]. An additional pixel layer, the “insertable B-layer” (IBL) [32] installed during the 2013-2015 shutdown between Run 1 and Run 2, is used in the 5.02 $\mathrm{TeV}$ measurements. The MBTS system detects charged particles over $2.1\lesssim |\eta |\lesssim 3.9$ using two hodoscopes of counters positioned at $z = \pm$3.6 m. The FCal consists of three sampling layers, longitudinal in shower depth, and covers $3.2<|\eta |< 4.9$. The ZDC are positioned at ±140 m from the IP, detecting neutrons and photons with $|\eta |>8.3$.

This analysis uses approximately 7 and 470 $\upmu \text {b}^{-1}$ of Pb+Pb data at $\sqrt{s_{\mathrm {NN}}}= 2.76$ and 5.02 $\mathrm{TeV}$, respectively, recorded by the ATLAS experiment at the LHC. The 2.76 TeV data were collected in 2010, while the 5.02 TeV data were collected in 2015.

The ATLAS trigger system [33] consists of a level-1 (L1) trigger implemented using a combination of dedicated electronics and programmable logic, and a high-level trigger (HLT) implemented in general-purpose processors. The trigger requires signals in both ZDC or either of the two MBTS counters. The ZDC trigger thresholds on each side are set below the maximum corresponding to a single neutron. A timing requirement based on signals from each side of the MBTS was imposed to remove beam backgrounds. This trigger selected 7 $\upmu \text {b}^{-1}$ and 22 $\upmu \text {b}^{-1}$ of minimum-bias Pb+Pb data at $\sqrt{s_{\mathrm {NN}}}= 2.76$ $\mathrm{TeV}$ and $\sqrt{s_{\mathrm {NN}}}= 5.02$ $\mathrm{TeV}$, respectively. To increase the number of recorded events from very central Pb+Pb collisions, a dedicated L1 trigger was used in 2015 to select events requiring the total transverse energy ($\Sigma E_{\text {T}}\,$) in the FCal to be more than 4.54 TeV. This ultra-central trigger sampled 470 $\upmu \hbox {b}^{-1}$ of Pb+Pb collisions at 5.02 TeV and was fully efficient for collisions with centrality 0–0.1% (see Sect. 4).

## Event and track selection

The offline event selection requires a reconstructed vertex with its *z* position satisfying $|Z_{\text {vtx}}\,|< 100$ mm. For the $\sqrt{s_{\mathrm {NN}}}= 2.76$ $\mathrm{TeV}$ Pb+Pb data, the selection also requires a time difference $|\Delta t| < 3$ ns between signals in the MBTS trigger counters on either side of the nominal centre of ATLAS to suppress non-collision backgrounds. A coincidence between the ZDC signals at forward and backward pseudorapidity is required to reject a variety of background processes such as elastic collisions and non-collision backgrounds, while maintaining high efficiency for inelastic processes. The fraction of events containing more than one inelastic interaction (pile-up) is estimated to be less than 0.1% at both collision energies. The pile-up contribution is studied by exploiting the correlation between the transverse energy $\Sigma E_{\text {T}}\,$ measured in the FCal or the number of neutrons $N_n$ in the ZDC and the number of tracks associated with a primary vertex $N_{\mathrm {ch}}^{\mathrm {rec}}$. Since the distribution of $\Sigma E_{\text {T}}\,$ or $N_n$ in events with pile-up is broader than that for the events without pile-up, pile-up events are suppressed by rejecting events with an abnormally large $\Sigma E_{\text {T}}\,$ or $N_n$ as a function of $N_{\mathrm {ch}}^{\mathrm {rec}}$.

The event centrality [34] is characterised by the $\Sigma E_{\text {T}}\,$ deposited in the FCal over the pseudorapidity range $3.2< |\eta | < 4.9$ using a calibration employing the electromagnetic calorimeters to set the energy scale [35]. The FCal $\Sigma E_{\text {T}}\,$ distribution is divided into a set of centrality intervals. A centrality interval refers to a percentile range, starting at 0% relative to the most central collisions. Thus the 0–5% centrality interval, for example, corresponds to the most central 5% of the events. The ultra-central trigger mentioned in Sect. 3 selects events in the 0–0.1% centrality interval with full efficiency. A Monte Carlo Glauber analysis [34, 36] is used to estimate the average number of participating nucleons, $N_{\mathrm {part}}$, for each centrality interval. The systematic uncertainty in $N_{\mathrm {part}}$ is less than 1% for centrality intervals in the range 0–20% and increases to 6% for centrality intervals in the range 70–80%. The Glauber model also provides a correspondence between the $\Sigma E_{\text {T}}\,$ distribution and sampling fraction of the total inelastic Pb+Pb cross section, allowing centrality percentiles to be set. For this analysis, a selection of collisions corresponding to 0–70% centrality is used to avoid diffraction or other processes that contribute to very peripheral collisions. Following the convention used in heavy-ion analyses, the centrality dependence of the results in this paper is presented as a function of $N_{\mathrm {part}}$.

Charged-particle tracks and primary vertices [37] are reconstructed from hits in the ID. Tracks are required to have $p_{\text {T}}\,>0.5$ GeV and $|\eta |<2.4$. For the 2.76 TeV data, tracks are required to have at least nine hits in the silicon detectors with no missing pixel hits and not more than one missing SCT hit, taking into account the presence of known dead modules. For the 5.02 TeV data, tracks are required to have at least two pixel hits, with the additional requirement of a hit in the first pixel layer when one is expected, at least eight SCT hits, and at most one missing hit in the SCT. In addition, for both datasets, the point of closest approach of the track is required to be within 1 mm of the primary vertex in both the transverse and longitudinal directions [38].

The efficiency, $\epsilon (p_{\text {T}}\,,\eta )$, of the track reconstruction and track selection criteria is evaluated using Pb+Pb Monte Carlo events produced with the HIJING event generator [39]. The generated particles in each event were rotated in azimuthal angle according to the procedure described in Ref. [40] to produce harmonic flow consistent with previous ATLAS measurements [5, 41]. The response of the detector was simulated using $\textsc {Geant}\,$4 [42, 43] and the resulting events are reconstructed with the same algorithms applied to the data. For the 5.02 TeV Pb+Pb data, the efficiency ranges from 75% at $\eta \approx 0$ to about 50% for $|\eta | > 2$ for charged particles with $p_{\text {T}}\,> 0.8$ GeV, falling by about 5% as $p_{\text {T}}\,$ is reduced to 0.5 GeV. The efficiency varies more strongly with $\eta$ and event multiplicity. For $p_{\text {T}}\,> 0.8$ GeV, it ranges from 75% at $\eta \approx 0$ to 50% for $|\eta | > 2$ in peripheral collisions, while it ranges from 71% at $\eta \approx 0$ to about 40% for $|\eta | > 2$ in central collisions. The tracking efficiency for the 2.76 TeV data has a similar dependence on $p_{\text {T}}\,$ and $\eta$. The efficiency is used in the particle weight, as described in Sect. 5. However, because the observables studied are ratios (see Sect. 2), uncertainties in detector and reconstruction efficiencies largely cancel. The rate of falsely reconstructed tracks (“fakes”) is also estimated and found to be significant only at $p_{\text {T}}\,<1$ GeV in central collisions, where its percentage per-track ranges from 2% at $|\eta |<1$ to 8% at the larger $|\eta |$. The fake rate drops rapidly for higher $p_{\text {T}}\,$ and towards more peripheral collisions. The fake rate is accounted for in the tracking efficiency correction following the procedure in Ref. [44].

## Data analysis

Measurement of the longitudinal flow dynamics requires the calculation of the flow vector ${\varvec{q}}_n$ via Eq. (1) in the ID and the FCal. The flow vector from the FCal serves as the reference ${\varvec{q}}_n(\eta _{\mathrm {ref}})$, while the ID provides the flow vector as a function of pseudorapidity ${\varvec{q}}_n(\eta )$.

In order to account for detector inefficiencies and non-uniformity, a particle weight for the $i{\text {th}}$-particle in the ID for the flow vector from Eq. (1) is defined as:

14 $$\begin{aligned} w_i^{\text {ID}}(\eta ,\phi ,p_{\text {T}}\,) = d_{\text {ID}} (\eta ,\phi )/\epsilon (\eta ,p_{\text {T}}\,), \end{aligned}$$

similar to the procedure in Ref. [44]. The determination of track efficiency $\epsilon (\eta ,p_{\text {T}}\,)$ is described in Sect. 4. The additional weight factor $d_{\text {ID}}(\eta ,\phi )$ corrects for variation of tracking efficiency or non-uniformity of detector acceptance as a function of $\eta$ and $\phi$. For a given $\eta$ interval of 0.1, the distribution in azimuthal bins, $N(\phi ,\eta )$, is built up from reconstructed charged particles summed over all events. The weight factor is then obtained as $d_{\text {ID}}(\eta ,\phi ) \equiv \left\langle N(\eta )\right\rangle /N(\phi ,\eta )$, where $\left\langle N(\eta )\right\rangle$ is the average of $N(\phi ,\eta )$. This “flattening” procedure removes most $\phi$-dependent non-uniformity from track reconstruction, which is important for any azimuthal correlation analysis. Similarly, the weight in the FCal for the flow vector from Eq. (1) is defined as:

15 $$\begin{aligned} w_i^{\text {FCal}}(\eta ,\phi ) = d_{\text {FCal}}(\eta ,\phi ) E_{{\text {T}},i}, \end{aligned}$$

where $E_{{\text {T}},i}$ is the transverse energy measured in the $\text {i}{\text {th}}$ tower in the FCal at $\eta$ and $\phi$. The azimuthal weight $d_{\text {FCal}}(\eta ,\phi )$ is calculated in narrow $\eta$ intervals in a similar way to what is done for the ID. It ensures that the $E_{\text {T}}$-weighted distribution, averaged over all events in a given centrality interval, is uniform in $\phi$. The flow vectors ${\varvec{q}}_n(\eta )$ and ${\varvec{q}}_n(\eta _{\mathrm {ref}})$ are further corrected by an event-averaged offset: ${\varvec{q}}_n -\left\langle {\varvec{q}}_n\right\rangle _{\text {evts}}$ [8].

The flow vectors obtained after these reweighting and offset procedures are used in the correlation analysis. The correlation quantities used in $r_{n|n;k}$ are calculated as:

16 $$\begin{aligned} \left\langle {\varvec{q}}_n^k (\eta ) {\varvec{q}}_n^{*k}(\eta _{\mathrm {ref}})\right\rangle\equiv & {} \left\langle {\varvec{q}}_n^k (\eta ) {\varvec{q}}_n^{*k}(\eta _{\mathrm {ref}})\right\rangle _{\text {s}}\nonumber \\&-\left\langle {\varvec{q}}_n^k (\eta ) {\varvec{q}}_n^{*k}(\eta _{\mathrm {ref}})\right\rangle _{\text {b}}, \end{aligned}$$

where subscripts “s” and “b” represent the correlator constructed from the same event (“signal”) and from the mixed-event (“background”), respectively. The mixed-event quantity is constructed by combining ${\varvec{q}}_n^k (\eta )$ from each event with ${\varvec{q}}_n^{*k}(\eta _{\mathrm {ref}})$ obtained in other events with similar centrality (within 1%) and similar $Z_{\text {vtx}}\,$ ($|\Delta Z_{\text {vtx}}\,|<5$ mm). The $\left\langle {\varvec{q}}_n^k (\eta ) {\varvec{q}}_n^{*k}(\eta _{\mathrm {ref}})\right\rangle _{\text {b}}$, which is typically more than two orders of magnitude smaller than the corresponding signal term, is subtracted to account for any residual detector non-uniformity effects that result from a correlation between different $\eta$ ranges.

For correlators involving flow vectors in two different $\eta$ ranges, mixed events are constructed from two different events. For example, the correlation for $r_{2,3|5}$ is calculated as:

17 $$\begin{aligned} \left\langle {\varvec{q}}_2 (\eta ) {\varvec{q}}_3 (\eta ) {\varvec{q}}_5^{*}(\eta _{\mathrm {ref}})\right\rangle\equiv & {} \left\langle {\varvec{q}}_2 (\eta ) {\varvec{q}}_3 (\eta ) {\varvec{q}}_5^{*}(\eta _{\mathrm {ref}})\right\rangle _{\text {s}}\nonumber \\&-\left\langle {\varvec{q}}_2 (\eta ) {\varvec{q}}_3 (\eta ) {\varvec{q}}_5^{*}(\eta _{\mathrm {ref}})\right\rangle _{\text {b}}. \end{aligned}$$

The mixed-event correlator is constructed by combining ${\varvec{q}}_2 (\eta ) {\varvec{q}}_3 (\eta )$ from one event with ${\varvec{q}}_5^{*}(\eta _{\mathrm {ref}})$ obtained in another event with similar centrality (within 1%) and similar $Z_{\text {vtx}}\,$ ($|\Delta Z_{\text {vtx}}\,|<5$ mm). On the other hand, for correlators involving more than two different $\eta$ ranges, mixed events are constructed from more than two different events, one for each unique $\eta$ range. One such example is $R_{n|n;2}$, for which each mixed event is constructed from four different events with similar centrality and $Z_{\text {vtx}}\,$.

Most correlators can be symmetrised. For example, in a symmetric system such as Pb+Pb collisions, the condition $\left\langle {\varvec{q}}_n^k (-\eta ) {\varvec{q}}_n^{*k}(\eta _{\mathrm {ref}})\right\rangle = \left\langle {\varvec{q}}_n^k (\eta ) {\varvec{q}}_n^{*k}(-\eta _{\mathrm {ref}})\right\rangle$ holds. So instead of Eq. (2), the actual measured observable is:

18 $$\begin{aligned} r_{n|n;k}(\eta ) = \frac{\left\langle {\varvec{q}}_n^k (-\eta ) {\varvec{q}}_n^{*k}(\eta _{\mathrm {ref}})+{\varvec{q}}_n^k (\eta ) {\varvec{q}}_n^{*k}(-\eta _{\mathrm {ref}})\right\rangle }{\left\langle {\varvec{q}}_n^k (\eta ){\varvec{q}}_n^{*k}(\eta _{\mathrm {ref}})+{\varvec{q}}_n^k (-\eta ){\varvec{q}}_n^{*k}(-\eta _{\mathrm {ref}})\right\rangle }. \end{aligned}$$

The symmetrisation procedure also allows further cancellation of possible differences between $\eta$ and $-\eta$ in the tracking efficiency or detector acceptance.

Table 1 gives a summary of the set of correlators measured in this analysis. The analysis is performed in intervals of centrality and the results are presented as a function of $\eta$ for $|\eta |<2.4$. The main results are obtained using 5.02 TeV Pb+Pb data. The 2010 2.76 TeV Pb+Pb data are statistically limited, and are used only to obtain $r_{n|n;1}$ and $R_{n|n;2}$ to compare with results obtained from the 5.02 TeV data and study the dependence on collision energy.

**Table 1:** The list of observables measured in this analysis

Observables	Pb+Pb datasets (Tev)
$r_{n|n;k} \;\;\;$for $n=2,3,4$ and $k=1$	2.76 and 5.02
$R_{n|n;2} \;\;$for $n=2,3$	2.76 and 5.02
$r_{n|n;k} \;\;\;$for $n=5$ and $k=1$	5.02
$r_{n|n;k} \;\;\;$for $n=2,3$ and $k=2$,3	5.02
$R_{n|n;2} \;\;$for $n=4$	5.02
$r_{2,2|4},\;r_{2,3|5},\;r_{2,3|2,3}$	5.02

**Fig. 2:**
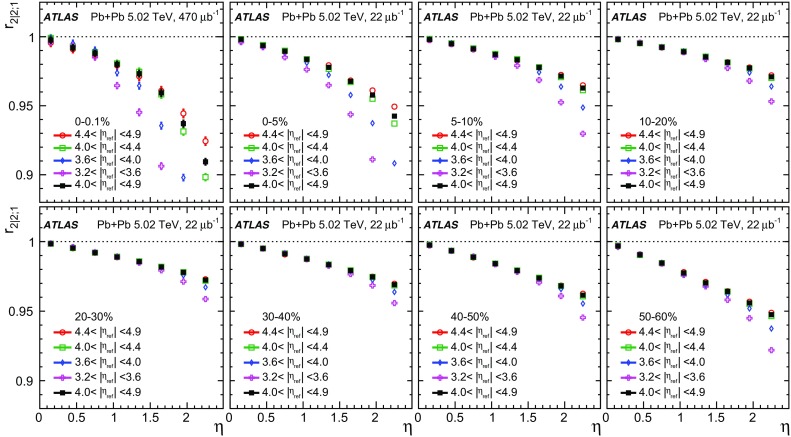
The $r_{2|2;1}(\eta )$ measured for several $\eta _{\mathrm {ref}}$ ranges. Each panel shows the results for one centrality range. The error bars are statistical only

**Fig. 3:**
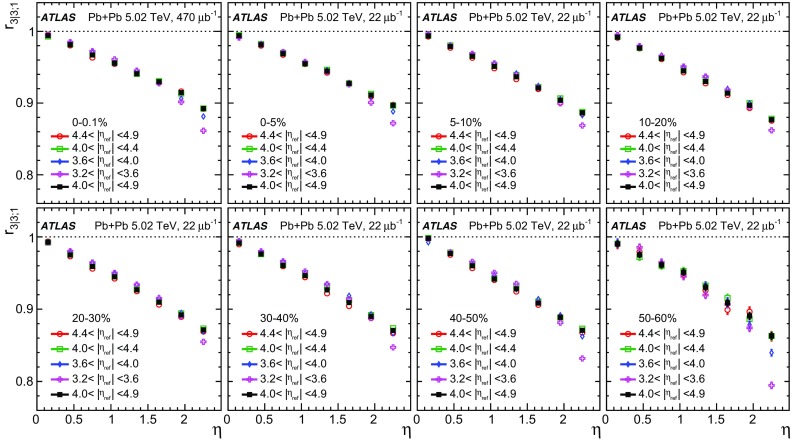
The $r_{3|3;1}(\eta )$ measured for several $\eta _{\mathrm {ref}}$ ranges. Each panel shows the results for one centrality range. The error bars are statistical only

Figures 2, 3 show the sensitivity of $r_{2|2;1}$ and $r_{3|3;1}$, respectively, to the choice of the range of $\eta _{\mathrm {ref}}$. A smaller $\eta _{\mathrm {ref}}$ value implies a smaller pseudorapidity gap between $\eta$ and $\eta _{\mathrm {ref}}$. The values of $r_{n|n;1}$ generally decrease with decreasing $\eta _{\mathrm {ref}}$, possibly reflecting the contributions from the dijet correlations [5]. However, such contributions should be reduced in the most central collisions due to large charged-particle multiplicity and jet-quenching [45] effects. Therefore, the decrease of $r_{n|n;1}$ in the most central collisions may also reflect the $\eta _{\mathrm {ref}}$ dependence of $F_{n;1}^{\text {r}}$, as defined in Eq. (3). In this analysis, the reference flow vector is calculated from $4.0<\eta _{\mathrm {ref}}<4.9$, which reduces the effect of dijets and provides good statistical precision. For this choice of $\eta _{\mathrm {ref}}$ range, $r_{2|2;1}$ and $r_{3|3;1}$ show a linear decrease as a function of $\eta$ in most centrality intervals, indicating a significant breakdown of factorisation. A similar comparison for $r_{4|4;1}$ can be found in the “Appendix”.

**Fig. 4:**
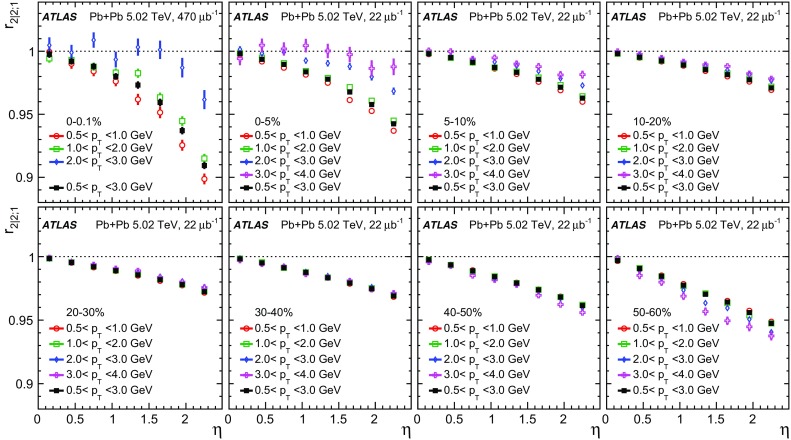
The $r_{2|2;1}(\eta )$ measured in several $p_{\text {T}}\,$ ranges. Each panel shows the results for one centrality range. The error bars are statistical only

**Fig. 5:**
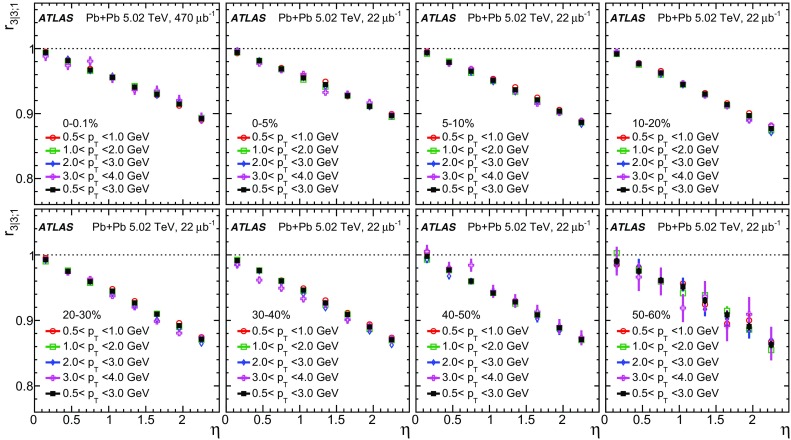
The $r_{3|3;1}(\eta )$ measured in several $p_{\text {T}}\,$ ranges. Each panel shows the results for one centrality range. The error bars are statistical only

Figures 4, 5 show $r_{2|2;1}$ and $r_{3|3;1}$ calculated for several $p_{\text {T}}\,$ ranges of the charged particles in the ID. A similar comparison for $r_{4|4;1}$ can be found in the “Appendix”. If the longitudinal-flow asymmetry and twist reflect global properties of the event, the values of $r_{n|n;1}$ should not depend strongly on $p_{\text {T}}\,$. Indeed no dependence is observed, except for $r_{2|2;1}$ in the most central collisions and very peripheral collisions. The behaviour in central collisions may be related to the factorisation breaking of the $v_2$ as a function of $p_{\text {T}}\,$ and $\eta$ [5, 19]. The behaviour in peripheral collisions is presumably due to increasing relative contributions from jets and dijets at higher $p_{\text {T}}\,$ and for peripheral collisions. Based on this, the measurements are performed using charged particles with $0.5<p_{\text {T}}\,<3$ GeV.

## Systematic uncertainties

Since all observables are found to follow an approximately linear decrease with $\eta$, i.e. $D(\eta ) \approx 1-c\eta$ for a given observable $D(\eta )$ where *c* is a constant, the systematic uncertainty is presented as the relative uncertainty for $1-D(\eta )$ at $\eta =1.2$, the mid-point of the $\eta$ range. The systematic uncertainties in this analysis arise from event mixing, track selection, and reconstruction efficiency. Most of the systematic uncertainties enter the analysis through the particle weights in Eqs. (14) and (15). In general, the uncertainties for $r_{n|n;k}$ increase with *n* and *k*, the uncertainties for $R_{n|n;2}$ increase with *n*, and all uncertainties are larger in the most central and more peripheral collisions. For $r_{2,3|2,3}$, $r_{2,2|4}$ and $r_{2,3|5}$, the uncertainties are significantly larger than for the other correlators. Each source is discussed separately below.

The effect of detector azimuthal non-uniformity is accounted for by the weight factor $d(\eta ,\phi )$ in Eqs. (14) and (15). The effect of reweighting is studied by setting the weight to unity and repeating the analysis. The results are consistent with the default (weighted) results within statistical uncertainties, so no additional systematic uncertainty is included. Possible residual detector effects for each observable are further removed by subtracting those obtained from mixed events as described in Sect. 5. Uncertainties due to the event-mixing procedure are estimated by varying the criteria for matching events in centrality and $z_{\mathrm {vtx}}$. The resulting uncertainty is in general found to be smaller than the statistical uncertainties. The event-mixing uncertainty for $r_{2|2;k}$ and $r_{3|3;k}$ is less than 1% for $k=1$ and changes to about 0.4–8% for $k=2$ and 0.6–10% for $k=3$, while the uncertainty for $r_{4|4;1}$ and $r_{5|5;1}$ is in the range 1.5–3% and 5–13%, respectively. The uncertainty for $R_{n|n;2}$ is 1.5–6% for $n=2$ and 3–14% for $n=3$. The uncertainties for $r_{2,3|2,3}$, $r_{2,2|4}$ and $r_{2,2|5}$ are typically larger: 1–4%, 1.5–16% and 3–15%.

The systematic uncertainty associated with the track quality selections is estimated by tightening or loosening the requirements on transverse impact parameter $|d_0|$ and longitudinal impact parameter $|z_0\sin \theta |$ used to select tracks. In each case, the tracking efficiency is re-evaluated and the analysis is repeated. The difference is observed to be larger in the most central collisions where the flow signal is smaller and the influence of falsely reconstructed tracks is higher. The difference is observed to be in the range 0.2–12% for $r_{2|2;k}$ and $r_{3|3;k}$, 1.1–2% for $r_{4|4;1}$, 3–6% for $r_{5|5;1}$, 0.5–13% for $R_{n|n;2}$, and 1–14% for $r_{2,3|2,3}$, $r_{2,2|4}$ and $r_{2,2|5}$.

From previous measurements [5, 6, 46], the $v_n$ signal has been shown to have a strong dependence on $p_{\text {T}}\,$ but relatively weak dependence on $\eta$. Therefore, a $p_{\text {T}}\,$-dependent uncertainty in the track reconstruction efficiency $\epsilon (\eta ,p_{\text {T}}\,)$ could affect the measured longitudinal flow correlation, through the particle weights. The uncertainty in the track reconstruction efficiency is due to differences in the detector conditions and known differences in the material between data and simulations. The uncertainty in the efficiency varies between 1% and 4%, depending on $\eta$ and $p_{\text {T}}\,$ [44]. The systematic uncertainty for each observable in Table 1 is evaluated by repeating the analysis with the tracking efficiency varied up and down by its corresponding uncertainty. For $r_{n|n;k}$ the uncertainties are in the range 0.1–2%, depending on *n* and *k*. For $R_{n|n;2}$ the uncertainties are in the range 0.1–1%. For $r_{2,3|2,3}$, $r_{2,2|4}$ and $r_{2,3|5}$, the uncertainties are in the range 0.1–2%.

Due to the finite energy resolution and energy scale uncertainty of the FCal, the ${\varvec{q}}_n(\eta _{\mathrm {ref}})$ calculated from the azimuthal distribution of the $E_{\text {T}}\,$ via Eqs. (1) and (15) differs from the true azimuthal distribution. However, since ${\varvec{q}}_n(\eta _{\mathrm {ref}})$ appears in both the numerator and the denominator of the correlators studied in this paper, most of the effects associated with the FCal $E_{\text {T}}\,$ response are expected to cancel out. Two cross-checks are also performed to study the influence of the FCal response. In the first cross-check, only the FCal towers with $E_{\text {T}}\,$ above the $50^{\mathrm {th}}$ percentile are used to calculate the ${\varvec{q}}_n(\eta _{\mathrm {ref}})$. The $|{\varvec{q}}_n(\eta _{\mathrm {ref}})|$ value is different from the default analysis, but the values of the correlators are found to be consistent. In the second cross-check, HIJING events with imposed flow (see Sect. 4) are used to study the FCal response. The ${\varvec{q}}_n(\eta _{\mathrm {ref}})$ is calculated using both the generated $E_{\text {T}}\,$ and the reconstructed $E_{\text {T}}\,$, and the resulting correlators are compared with each other. The results are found to be consistent. Accordingly, no additional systematic uncertainty is added for the FCal response.

The systematic uncertainties from the different sources described above are added in quadrature to give the total systematic uncertainty for each observable. They are listed in Tables 2, 3 and 4.

**Table 2:** Systematic uncertainties in percent for $1-r_{2|2;k}$ and $1-r_{3|3;k}$ at $\eta =1.2$ in selected centrality intervals

	1$-r_{2|2;1}$			1$-r_{2|2;2}$			1$-r_{2|2;3}$		
	0–5%	20–30%	40–50%	0–5%	20–30%	40–50%	0–5%	20–30%	40–50%
Event mixing (%)	0.8	0.2	0.3	2.2	0.4	0.6	6.0	0.6	2.1
Track selections (%)	0.4	0.3	0.2	1.5	0.4	0.9	9.4	1.0	2.4
Reco. efficiency (%)	0.3	0.1	0.1	0.4	0.1	0.1	0.9	0.1	0.1
Total (%)	1.0	0.4	0.4	2.7	0.6	1.1	12	1.2	3.2

	1$-r_{3|3;1}$			1$-r_{3|3;2}$			1$-r_{3|3;3}$		
	0–5%	20–30%	40–50%	0–5%	20–30%	40–50%	0–5%	20–30%	
Event mixing (%)	0.6	0.4	0.9	2.2	1.2	7.9	7.0	9.5	
Track selections (%)	0.6	0.2	0.6	2.5	0.7	4.4	12	10	
Reco. efficiency (%)	0.1	0.1	0.1	0.4	0.2	0.9	1.1	1.5	
Total (%)	0.9	0.5	1.1	3.4	1.5	9.1	14	14

**Table 3:** Systematic uncertainties in percent for $1-R_{2|2;2}$, $1-R_{3|3;2}$, $1-r_{4|4;1}$ and $1-r_{5|5;1}$ at $\eta =1.2$ in selected centrality intervals

	1$-R_{2|2;2}$			1$-R_{3|3;2}$		
	0–5%	20–30%	40–50%	0–5%	20–30%	40–50%
Event mixing (%)	6.1	1.5	1.5	4.6	2.9	14
Track selections (%)	3.5	0.4	0.7	2.0	3.2	13
Reco. efficiency(%)	0.2	0.1	0.1	0.1	0.2	0.5
Total (%)	7.1	1.6	1.7	5.1	4.4	20

	1$-r_{4|4;1}$			1$-r_{5|5;1}$		
	0–5%	20–30%	40–50%	0–5%	20–30%	40–50%
Event mixing (%)	1.8	1.5	2.7	13	5.1	9.8
Track selections (%)	1.5	1.1	2.0	6.3	3.6	4.6
Reco. efficiency(%)	0.3	0.3	0.6	2.2	1.6	1.3
Total (%)	2.4	1.9	3.5	15	6.5	11

**Table 4:** Systematic uncertainties in percent for $1-r_{2,3|2,3}$, $1-r_{2,2|4}$ and $1-r_{2,3|5}$ at $\eta =1.2$ in selected centrality intervals

	1$-r_{2,3|2,3}$			1$-r_{2,2|4}$			1$-r_{2,3|5}$		
	0–5%	20–30%	40–50%	0–5%	20–30%	40–50%	0–5%	20–30%	40–50%
Event mixing (%)	4.1	1.7	3.2	16	1.5	2.4	15	3.4	7.8
Track selections (%)	1.4	0.5	2.0	12	1.6	1.5	14	2.0	7.4
Reco. efficiency (%)	0.1	0.0	0.1	1.6	0.1	0.1	1.2	0.1	0.5
Total (%)	4.4	1.8	3.8	21	2.2	2.9	21	4.0	11

## Results

The presentation of the results is structured as follows. Section 7.1 presents the results for $r_{n|n;1}$ and $R_{n|n;2}$ and the comparison between the two collision energies. Section 7.2 shows the results for $r_{n|n;k}$ for $k>1$. The scaling relation from Eq. (4) is tested and the contributions from $v_n$ FB asymmetry and event-plane twist are estimated. Results for the mixed-harmonic correlators, Eqs. (7)–(9), are presented in Sect. 7.3 and checked for compatibility with the hydrodynamical picture. The measurements are performed using charged particles with $0.5<p_{\text {T}}\,<3$ GeV, and the reference flow vector is calculated with $4.0<|\eta _{\mathrm {ref}}|<4.9$. Most results are shown for the $\sqrt{s_{\mathrm {NN}}}=5.02$ TeV Pb+Pb dataset, which has better statistical precision. The results for the $\sqrt{s_{\mathrm {NN}}}=2.76$ TeV Pb+Pb dataset are shown only for $r_{n|n;1}$ and $R_{n|n;2}$.

### $r_{n|n;1}$ and $R_{n|n;2}$ at two collision energies

**Fig. 6:**
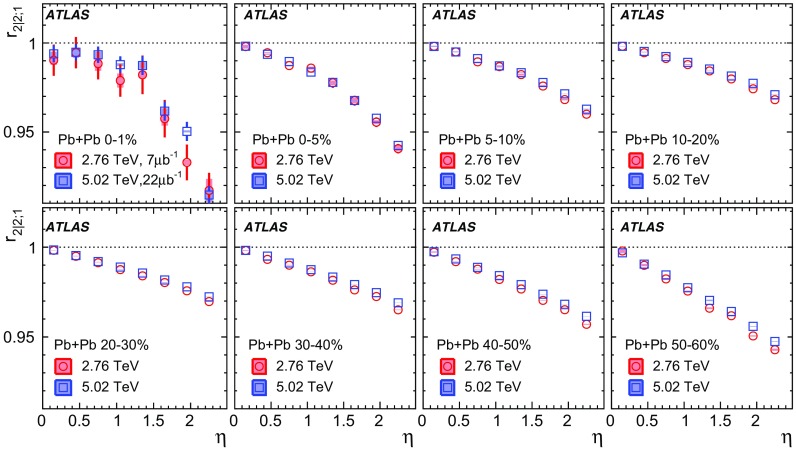
The $r_{2|2;1}(\eta )$ compared between the two collision energies. Each panel shows results from one centrality interval. The error bars and shaded boxes are statistical and systematic uncertainties, respectively

Figure 6 shows $r_{2|2;1}$ in various centrality intervals at the two collision energies. The correlator shows a linear decrease with $\eta$, except in the most central collisions. The decreasing trend is weakest around the 20–30% centrality range, and is more pronounced in both more central and more peripheral collisions. This centrality dependence is the result of a strong centrality dependence of the $v_2$ associated with the average elliptic geometry [47]. The decreasing trend at $\sqrt{s_{\mathrm {NN}}}=2.76$ TeV is slightly stronger than that at $\sqrt{s_{\mathrm {NN}}}=5.02$ TeV, which is expected as the collision system becomes less boost-invariant at lower collision energy [24].

**Fig. 7:**
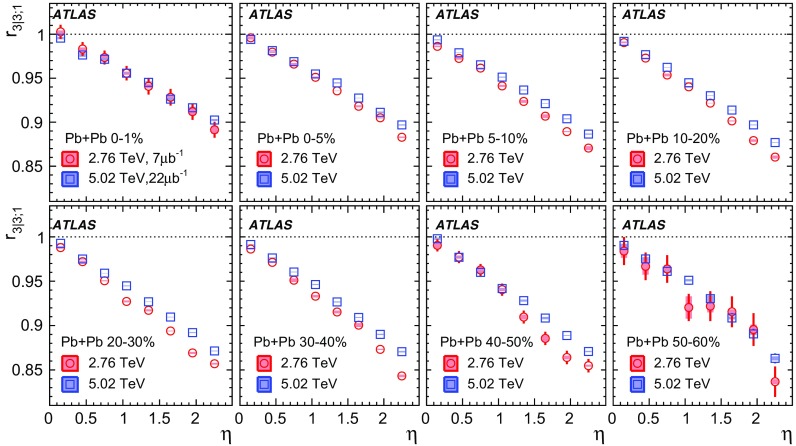
The $r_{3|3;1}(\eta )$ compared between the two collision energies. Each panel shows results from one centrality interval. The error bars and shaded boxes are statistical and systematic uncertainties, respectively

**Fig. 8:**
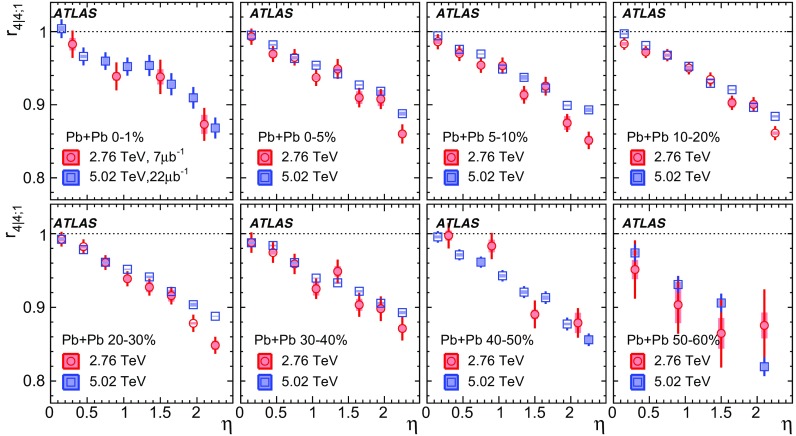
The $r_{4|4;1}(\eta )$ compared between the two collision energies. Each panel shows results from one centrality interval. The error bars and shaded boxes are statistical and systematic uncertainties, respectively

Figures 7 and 8 show the results for $r_{3|3;1}$ and $r_{4|4;1}$, respectively, at the two collision energies. A linear decrease as a function of $\eta$ is observed for both correlators, and the rate of the decrease is approximately independent of centrality. This centrality independence could be due to the fact that $v_3$ and $v_4$ are driven mainly by fluctuations in the initial state. The rate of the decrease is also observed to be slightly stronger at lower collision energy.

The decreasing trend of $r_{n|n;1}$ for $n=2$–4 in Figs. 6, 7 and 8 indicates significant breakdown of the factorisation of two-particle flow harmonics into those between different $\eta$ ranges. However, the size of the factorisation breakdown depends on the harmonic order *n*, collision centrality, and collision energy. The results have also been compared with those from the CMS Collaboration [19], with the $\eta _{\mathrm {ref}}$ chosen to be $4.4<|\eta _{\mathrm {ref}}|<4.9$ to match the CMS choice of $\eta _{\mathrm {ref}}$. The two results agree very well with each other, and details are shown in the “Appendix”.

**Fig. 9:**
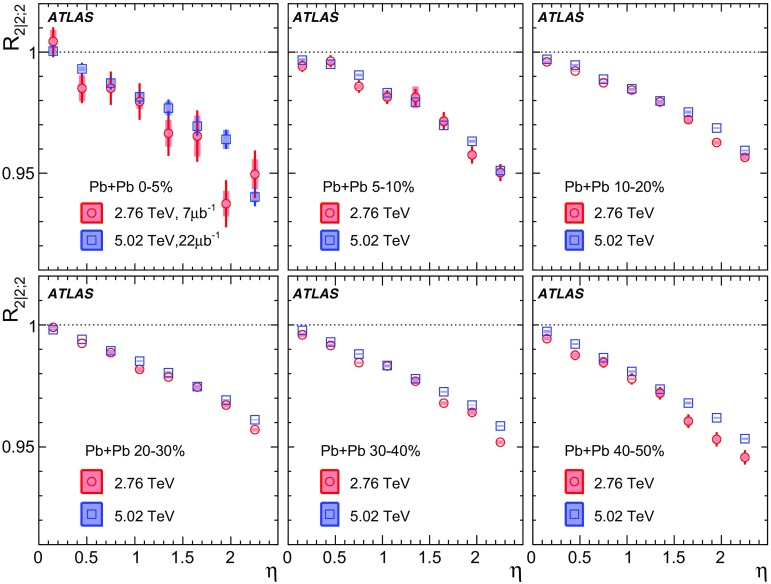
The $R_{2|2;2}(\eta )$ compared between the two collision energies. Each panel shows results from one centrality interval. The error bars and shaded boxes are statistical and systematic uncertainties, respectively

**Fig. 10:**
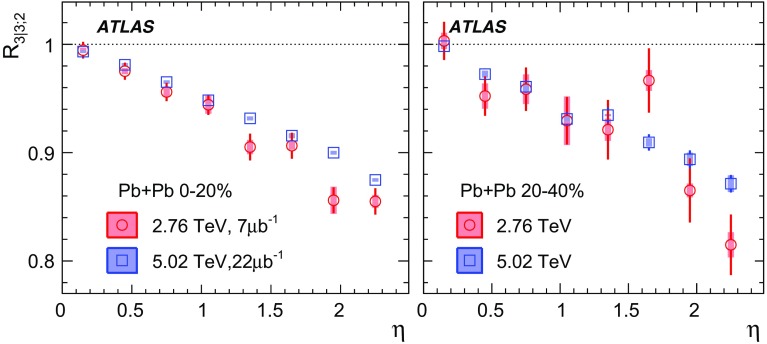
The $R_{3|3;2}(\eta )$ compared between the two collision energies. Each panel shows results from one centrality interval. The error bars and shaded boxes are statistical and systematic uncertainties, respectively

Figures 9 and 10 show $R_{2|2;2}$ and $R_{3|3;2}$ in several centrality intervals. Both observables follow a linear decrease with $\eta$ and the decreasing trends are stronger at lower collision energy.

The measured $r_{n|n;k}$ and $R_{n|n;2}$ are parameterised with linear functions,

19 $$\begin{aligned} r_{n|n;k} = 1-2F_{n;k}^{\text {r}}\;\eta ,\;\;R_{n|n;2} = 1-2F_{n;2}^{\text {R}}\;\eta , \end{aligned}$$

where the slope parameters are calculated as linear-regression coefficients,

20 $$\begin{aligned} F_{n;k}^{\text {r}}= & {} \frac{\sum _i(1-r_{n|n;k}(\eta _i))\eta _i}{2\sum _i\eta _i^2},\nonumber \\ F_{n;2}^{\text {R}}= & {} \frac{\sum _i(1-R_{n|n;2}(\eta _i))\eta _i}{2\sum _i\eta _i^2}, \end{aligned}$$

which characterise the average $\eta$-weighted deviation of $r_{n|n;1}(\eta )$ and $R_{n|n;2}(\eta )$ from unity. The sum runs over all data points. If $r_{n|n;k}$ and $R_{n|n;2}$ are a linear function in $\eta$, the linear-regression coefficients are equivalent to a fit to Eq. (19). However, these coefficients are well defined even if the observables have significant nonlinear behaviour, which is the case for $r_{2|2;k}$ and $R_{2|2;2}$ in the 0–20% centrality range.

**Fig. 11:**
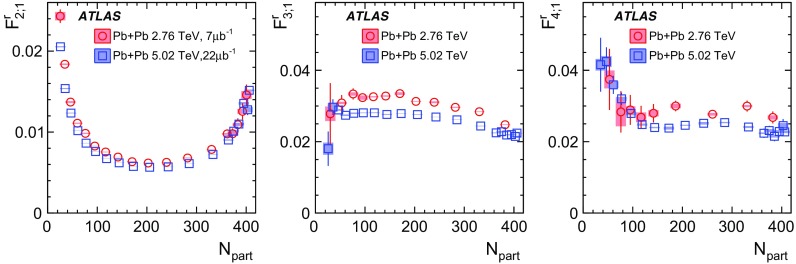
Centrality dependence of $F_{2;1}^{\text {r}}$ (left panel), $F_{3;1}^{\text {r}}$ (middle panel) and $F_{4;1}^{\text {r}}$ (right panel) for Pb+Pb at 2.76 TeV (circles) and 5.02 TeV (squares). The error bars and shaded boxes are statistical and systematic uncertainties, respectively. The widths of the centrality intervals are not fixed but are optimised to reduce the uncertainty

**Fig. 12:**
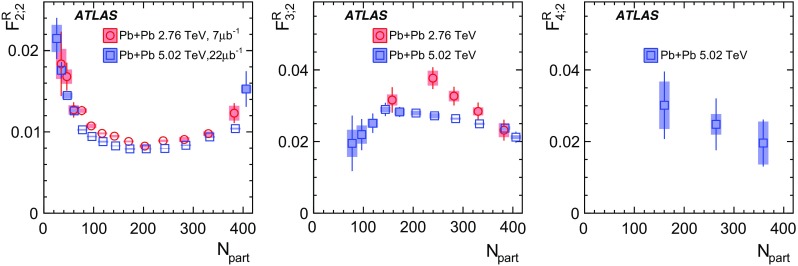
Centrality dependence of $F_{2;2}^{\text {R}}$ (left panel), $F_{3;2}^{\text {R}}$ (middle panel) and $F_{4;2}^{\text {R}}$ (right panel) for Pb+Pb at 2.76 TeV (circles) and 5.02 TeV (squares). The error bars and shaded boxes are statistical and systematic uncertainties, respectively. The widths of the centrality intervals are not fixed but are optimised to reduce the uncertainty

**Fig. 13:**
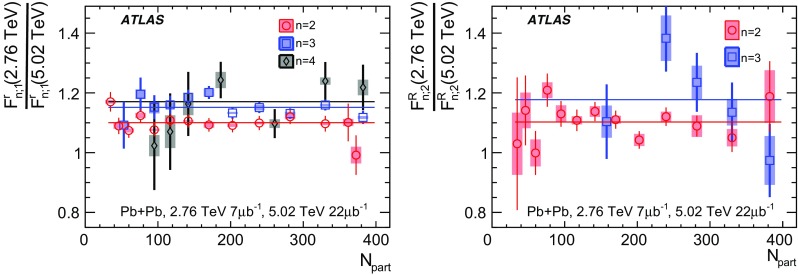
Centrality dependence of ratio of $F_{n;1}^{\text {r}}$ values (left panel) and $F_{n;2}^{\text {R}}$ values (right panel) at 2.76 and 5.02 TeV. The lines indicate the average values in the range $30<N_{\mathrm {part}}<400$, with the results and fit uncertainties given by Table 5. The error bars and shaded boxes are statistical and systematic uncertainties, respectively

The extracted slope parameters $F_{n;1}^{\text {r}}$ and $F_{n;2}^{\text {R}}$ are plotted as a function of centrality in terms of $N_{\mathrm {part}}$, in Figs. 11 and 12, respectively. The values of $F_{2;1}^{\text {r}}$ and $F_{2;2}^{\text {R}}$ first decrease and then increase as a function of increasing $N_{\mathrm {part}}$. The larger values in central and peripheral collisions are related to the fact that $v_2$ is more dominated by the initial geometry fluctuations. The slopes for higher-order harmonics are significantly larger. As a function of $N_{\mathrm {part}}$, a slight decrease in $F_{3;1}^{\text {r}}$ and $F_{3;2}^{\text {R}}$ is observed for $N_{\mathrm {part}}>200$, as well as an increase in $F_{4;1}^{\text {r}}$ for $N_{\mathrm {part}}<100$. The values of $F_{n;1}^{\text {r}}$ and $F_{n;2}^{\text {R}}$ are larger with decreasing $\sqrt{s_{\mathrm {NN}}}$, as the rapidity profile of the initial state is more compressed due to smaller beam rapidity $y_{\mathrm {beam}}$ at lower $\sqrt{s_{\mathrm {NN}}}$. This energy dependence has been predicted for $F_{n;1}^{\text {r}}$ in hydrodynamic model calculations [24], and it is quantified in Fig. 13 via the ratio of $F_{2;1}^{\text {r}}$ values and of $F_{2;2}^{\text {R}}$ values at the two energies. The weighted averages of the ratios calculated in the range $30<N_{\mathrm {part}}<400$ are given in Table 5. Compared to $\sqrt{s_{\mathrm {NN}}}=5.02$ TeV, the values of $F_{2;1}^{\text {r}}$ and $F_{2;2}^{\text {R}}$ at $\sqrt{s_{\mathrm {NN}}}=2.76$ TeV are about 10% higher, and the values of $F_{3;1}^{\text {r}}$ and $F_{4;1}^{\text {r}}$ are about 16% higher.

**Fig. 14:**
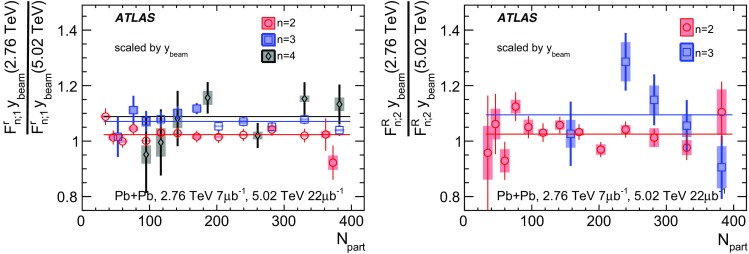
Centrality dependence of ratio of $\hat{F}_{n;1}^{\text {r}} \equiv F_{n;1}^{\text {r}}y_{\mathrm {beam}}$ values (left panel) and $\hat{F}_{n;2}^{\text {R}}\equiv F_{n;2}^{\text {R}}y_{\mathrm {beam}}$ values (right panel) at 2.76 and 5.02 TeV. The lines indicate the average values in the range $30<N_{\mathrm {part}}<400$, with the results and fit uncertainties given by Table 5. The error bars and shaded boxes are statistical and systematic uncertainties, respectively

If the change of correlators with $\sqrt{s_{\mathrm {NN}}}$ were entirely due to the change of $y_{\mathrm {beam}}$, then the correlators would be expected to follow a universal curve when they are rescaled by $y_{\mathrm {beam}}$, i. e. $r_{n|n;k}(\eta /y_{\mathrm {beam}})$ and $R_{n|n;2}(\eta /y_{\mathrm {beam}})$ should not depend on $\sqrt{s_{\mathrm {NN}}}$. In this case, the slopes parameters multiplified by the beam rapidity, $\hat{F}_{n;1}^{\text {r}}\equiv F_{n;1}^{\text {r}}y_{\mathrm {beam}}$ and $\hat{F}_{n;2}^{\text {R}}\equiv F_{n;2}^{\text {R}}y_{\mathrm {beam}}$, should not depend on $\sqrt{s_{\mathrm {NN}}}$. The beam rapidity is $y_{\mathrm {beam}}=7.92$ and 8.52 for $\sqrt{s_{\mathrm {NN}}}=2.76$ and 5.02 TeV, respectively, which leads to a 7.5% reduction in the ratio. Figure 14 shows the ratio of $\hat{F}_{2;1}^{\text {r}}$ values and of $\hat{F}_{2;2}^{\text {R}}$ values at the two energies, and the weighted averages of the ratios calculated in the range $30<N_{\mathrm {part}}<400$ are given in Table 5. The $y_{\mathrm {beam}}$-scaling accounts for a large part of the $\sqrt{s_{\mathrm {NN}}}$ dependence. Compared to $\sqrt{s_{\mathrm {NN}}}=5.02$ TeV, the values of $\hat{F}_{2;1}^{\text {r}}$ and $\hat{F}_{2;2}^{\text {R}}$ at $\sqrt{s_{\mathrm {NN}}}=2.76$ TeV are about 3% higher, and the values of $\hat{F}_{3;1}^{\text {r}}$ and $\hat{F}_{4;1}^{\text {r}}$ are about 8% higher, so this level of difference remains after accounting for the change in the beam rapidity.

**Table 5:** Results of the fits to the ratio of $F_{n;1}^{\text {r}}$, $F_{n;2}^{\text {R}}$, $\hat{F}_{n;1}^{\text {r}}\equiv F_{n;1}^{\text {r}}y_{\mathrm {beam}}$ and $\hat{F}_{n;2}^{\text {R}}\equiv F_{n;2}^{\text {R}}y_{\mathrm {beam}}$ values at the two energies in the range $30<N_{\mathrm {part}}<400$ shown in Figs. 13 and 14. The uncertainties include both statistical and systematic uncertainties

	$n=2$	$n=3$	$n=4$
$F_{n;1}^{\text {r}}(2.76~{\text {TeV}})\phantom {\int }{/}F_{n;1}^{\text {r}}(5.02~{\text {TeV}})$	$1.100\pm 0.010$	$1.152\pm 0.011$	$1.17\pm 0.036$
$F_{n;2}^{\text {R}}(2.76~{\text {TeV}})\phantom {\int }{/}F_{n;2}^{\text {R}}(5.02~{\text {TeV}})$	$1.103\pm 0.026$	$1.18\pm 0.08$	–
$\hat{F}_{n;1}^{\text {r}}(2.76~{\text {TeV}})\phantom {\int }{/}\hat{F}_{n;1}^{\text {r}}(5.02~{\text {TeV}})$	$1.023\pm 0.009$	$1.071\pm 0.010$	$1.088\pm 0.033$
$\hat{F}_{n;2}^{\text {R}}(2.76~{\text {TeV}})\phantom {\int }{/}\hat{F}_{n;2}^{\text {R}}(5.02~{\text {TeV}})$	$1.025\pm 0.024$	$1.10\pm 0.07$	–

### Higher-order moments

The longitudinal correlations of higher-order moments of harmonic flow carry information about the EbyE flow fluctuations in pseudorapidity. In the simple model described in Ref. [20], the decrease in $r_{n|n;k}$ is expected to scale with *k* as given by Eq. (4).

**Fig. 15:**
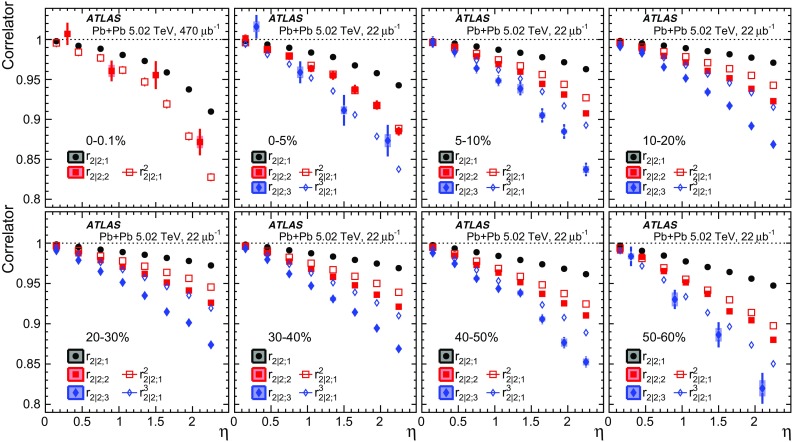
The $$r_{2|2;k}$$ for $$k=1$$–3 compared with $$r_{2|2;1}^k$$ for $$k = $$2–3 in various centrality intervals for Pb+Pb collisions at 5.02 TeV. The error bars and shaded boxes are statistical and systematic uncertainties, respectively. The data points for $$k=2$$ or 3 in some centrality intervals are rebinned to reduce the uncertainty

Figure 15 compares the results for $r_{2|2;k}$ for $k=1$–3 (solid symbols) with $r_{2|2;1}^k$ for $k=2$–3 (open symbols). The data follow the scaling relation from Eq. (4) in the most central collisions (0–5% centrality) where $v_2$ is driven by the initial-state fluctuations. In other centrality intervals, where the average geometry is more important for $v_2$, the $r_{2|2;k}$ ($k=2$ and 3) data show stronger decreases with $\eta$ than $r_{2|2;1}^k$.

**Fig. 16:**
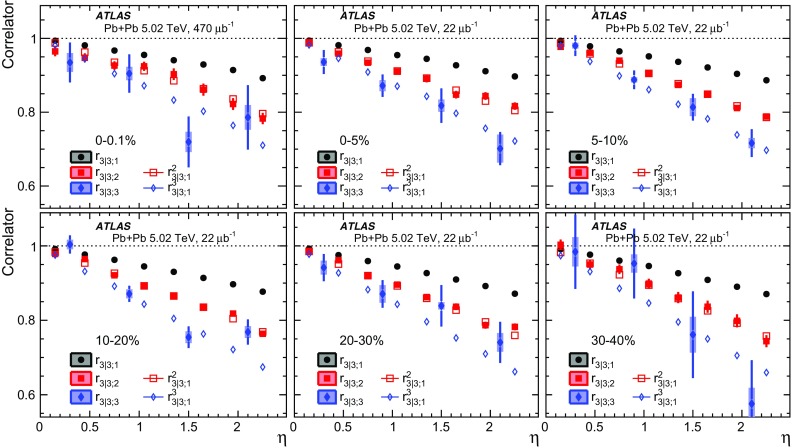
The $r_{3|3;k}$ for $k=1$–3 compared with $r_{3|3;1}^k$ for $k=2$–3 in various centrality intervals for Pb+Pb collisions at 5.02 TeV. The error bars and shaded boxes are statistical and systematic uncertainties, respectively. The data points for $k=2$ or 3 in some centrality intervals are rebinned to reduce the uncertainty

A similar study is performed for third-order harmonics, and the results are shown in Fig. 16. The data follow approximately the scaling relation Eq. (4) in all centrality intervals.

**Fig. 17:**
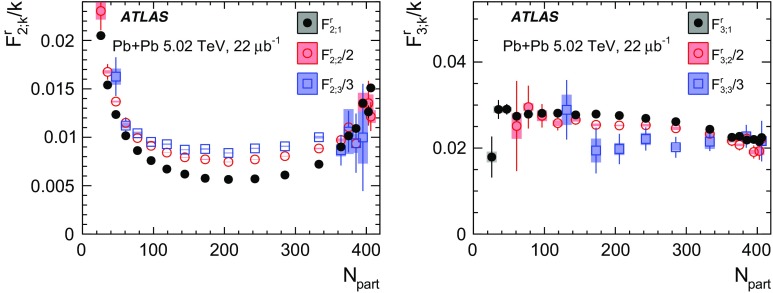
The values of $F_{n;k}^{\text {r}}/k$ for $k=1$,2 and 3 for $n=2$ (left panel) and $n=3$ (right panel), respectively. The error bars and shaded boxes are statistical and systematic uncertainties, respectively. The widths of the centrality intervals are not fixed but are optimised to reduce the uncertainty

To quantify the difference between $r_{n|n;k}$ and $r_{n|n;1}^k$, the slopes ($F_{n;k}^{\text {r}}$) of $r_{n|n;k}$ are calculated via Eqs. (19) and (20). The scaled quantities, $F_{n;k}^{\text {r}}/k$, are then compared with each other as a function of centrality in Fig. 17. For second-order harmonics, the data show clearly that over most of the centrality range $F_{2;3}^{\text {r}}/3>F_{2;2}^{\text {r}}/2>F_{2;1}^{\text {r}}$, implying $F_{2;k}^{\text {r}}>kF_{2;1}^{\text {r}}$. However, for the most central and most peripheral collisions the quantities approach each other. On the other hand, a slightly opposite trend for the third-order harmonics, $F_{3;3}^{\text {r}}/3\lesssim F_{3;2}^{\text {r}}/2\lesssim F_{3;1}^{\text {r}}$, i.e. $F_{3;k}^{\text {r}}\lesssim kF_{3;1}^{\text {r}}$, is observed in mid-central collisions ($150<N_{\mathrm {part}}<350$).

**Fig. 18:**
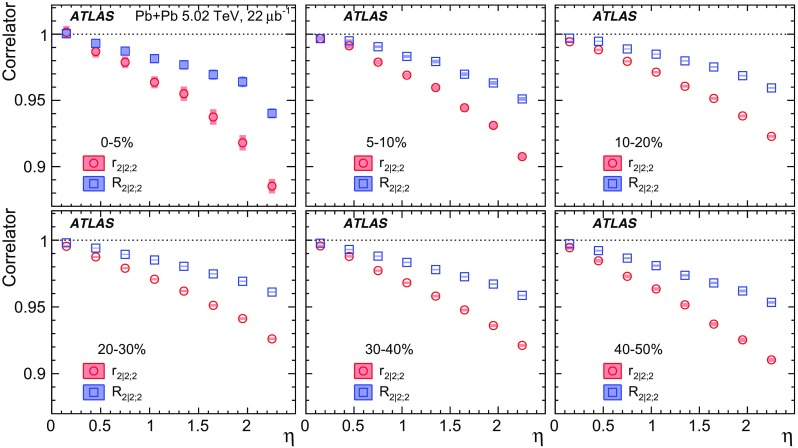
The $r_{2|2;2}(\eta )$ and $R_{2|2;2}(\eta )$ in various centrality intervals for Pb+Pb collisions at 5.02 TeV. The error bars and shaded boxes are statistical and systematic uncertainties, respectively

**Fig. 19:**
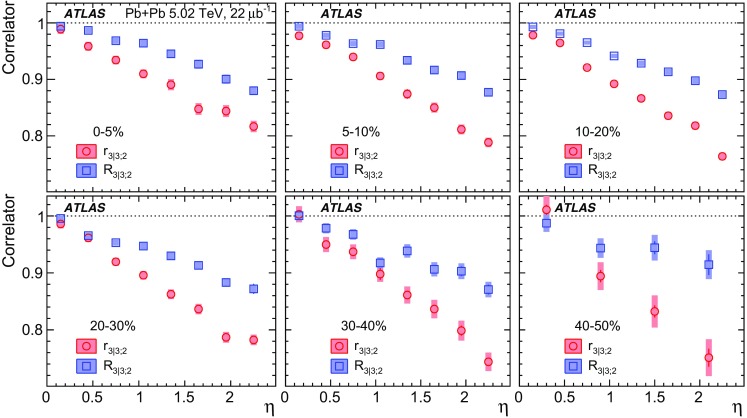
The $r_{3|3;2}(\eta )$ and $R_{3|3;2}(\eta )$ in various centrality intervals for Pb+Pb collisions at 5.02 TeV. The error bars and shaded boxes are statistical and systematic uncertainties, respectively. The data points in 40–50% centrality interval are rebinned to reduce the uncertainty

Figures 18 and 19 compare the $r_{n|n;2}$ with $R_{n|n;2}$ for $n=2$ and $n=3$, respectively. The decorrelation of $R_{n|n;2}$ is significantly weaker than that for the $r_{n|n;2}$. This is because the $R_{n|n;2}$ is mainly affected by the event-plane twist effects, while the $r_{n|n;2}$ receives contributions from both FB asymmetry and event-plane twist [20].

Following the discussion in Sect. 2, Eqs. (3) and (6), the measured $F_{n;2}^{\text {r}}$ and $F_{n;2}^{\text {R}}$ values can be used to estimate the separate contributions from FB asymmetry and event-plane twist, $F_{n;2}^{\text {asy}}$ and $F_{n;2}^{\text {twi}}$, respectively, via the relation:

21 $$\begin{aligned} F_{n;2}^{\text {twi}} = F_{n;2}^{\text {R}},\;\;\; F_{n;2}^{\text {asy}} = F_{n;2}^{\text {r}}- F_{n;2}^{\text {R}}. \end{aligned}$$

**Fig. 20:**
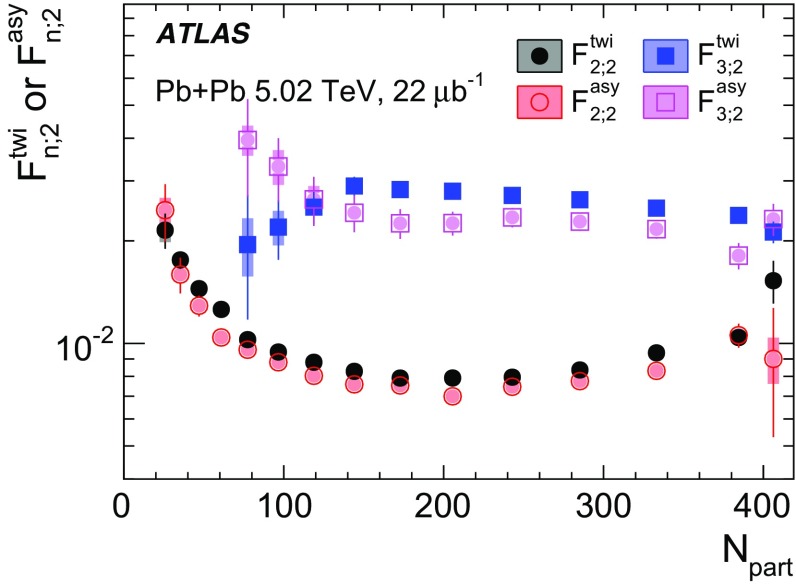
The estimated event-plane twist component $F_{n;2}^{\text {twi}}$ and FB asymmetry component $F_{n;2}^{\text {asy}}$ as a function of $N_{\mathrm {part}}$ for $n=2$ and 3 for Pb+Pb collisions at 5.02 TeV. The error bars and shaded boxes are statistical and systematic uncertainties, respectively

The results are shown in Fig. 20. The contributions from the two components are similar to each other for $n=2$, for which the harmonic flow arises primarily from the average collision shape, as well as for $n=3$, for which the harmonic flow is driven mainly by fluctuations in the initial geometry.

### Mixed-harmonics correlation

**Fig. 21:**
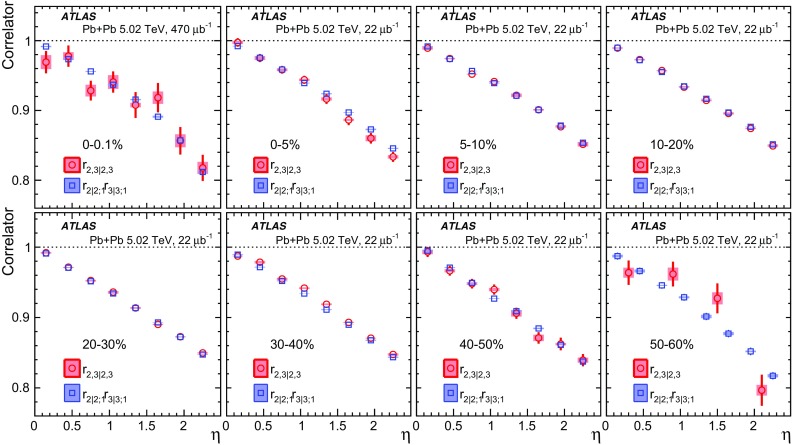
The $r_{2,3|2,3}$ (circles) and $r_{2|2;1}r_{3|3;1}$ (squares) as a function of $\eta$ for several centrality intervals. The error bars and shaded boxes are statistical and systematic uncertainties, respectively. The $r_{2,3|2,3}$ data in the 50–60% centrality interval are rebinned to reduce the uncertainty

**Fig. 22:**
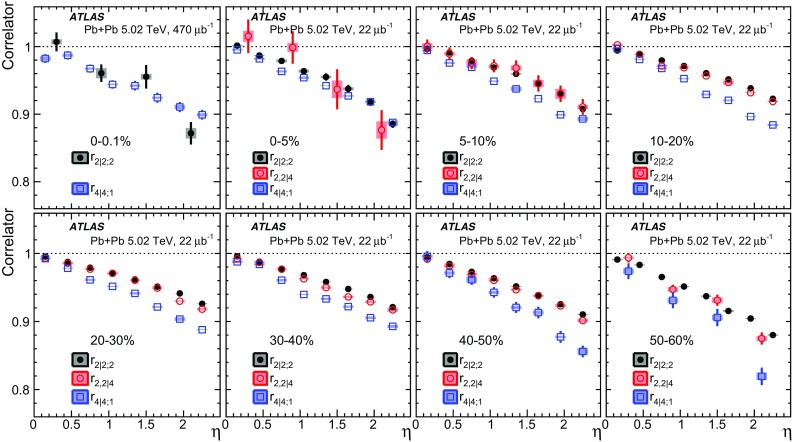
Comparison of $r_{2|2;2}$, $r_{2,2|4}$ and $r_{4|4;1}$ for several centrality intervals. The error bars and shaded boxes are statistical and systematic uncertainties, respectively. The data points in some centrality intervals are rebinned to reduce the uncertainty

Figure 21 compares the $r_{2,3|2,3}$ with the product of $r_{2|2;1}$ and $r_{3|3;1}$. The data show that they are consistent with each other, suggesting the previously observed anticorrelation beween $v_2$ and $v_3$ is a property of the entire event [9, 48], and that longitudinal fluctuations of $v_2$ and $v_3$ are uncorrelated. Figure 22 compares $r_{2|2;2}$ with the mixed-harmonic correlator $r_{2,2|4}$, as well as $r_{4|4;1}$. As discussed in Sect. 2 in the context of the first relation in Eq. (10), if the linear and non-linear components of $v_4$ in Eq. (10) are uncorrelated, then $r_{2,2|4}$ would be expected to be similar to $r_{2|2;24}$. This is indeed confirmed by the comparisons of the $\eta$ and centrality dependence of $r_{2|2;2}$ and $r_{2,2|4}$ in Fig. 22. Figure 22 also shows that the $\eta$ dependence for $r_{4|4;1}$ is stronger than for $r_{2|2;2}$ in all centrality intervals, suggesting that the decorrelation effects are stronger for the linear component of $v_4$ than for the nonlinear component (see Eq. (12)).

**Fig. 23:**
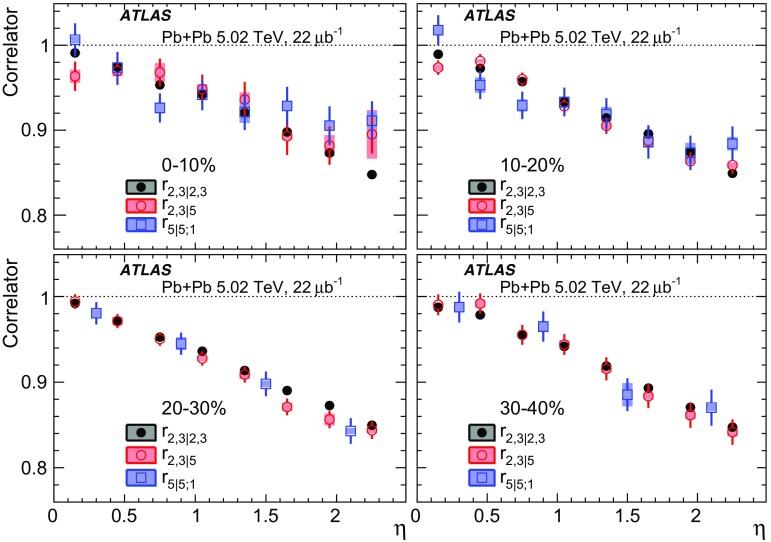
Comparison of $r_{2,3|2,3}$, $r_{2,3|5}$ and $r_{5|5;1}$ for several centrality intervals. The error bars and shaded boxes are statistical and systematic uncertainties, respectively. The $r_{5|5;1}$ data in some centrality intervals are rebinned to reduce the uncertainty

A similar study of the influence of the linear and nonlinear effects for $v_5$ was also performed, and results are shown in Fig. 23. The three observables $r_{2,3|2,3}$, $r_{2,3|5}$, and $r_{5|5;1}$ show similar values in all centrality intervals, albeit with large statistical uncertainties.

The decorrelations shown in Figs. 21, 22 and 23 can be quantified by calculating the slopes of the distributions in each centrality interval and presenting the results as a function of centrality. Following the example for $r_{n|n;k}$, the slopes for the mixed-harmonic correlators are obtained via the linear regression procedure of Eqs. (19) and (20):

22 $$\begin{aligned} r_{2,3|2,3} = 1-2F_{2,3|2,3}^{\text {r}}\;\eta ,\;\;r_{2,2|4} = 1-2F_{2,2|4}^{\text {r}}\;\eta ,\;\;r_{2,3|5} = 1-2F_{2,3|5}^{\text {r}}\;\eta . \end{aligned}$$

**Fig. 24:**
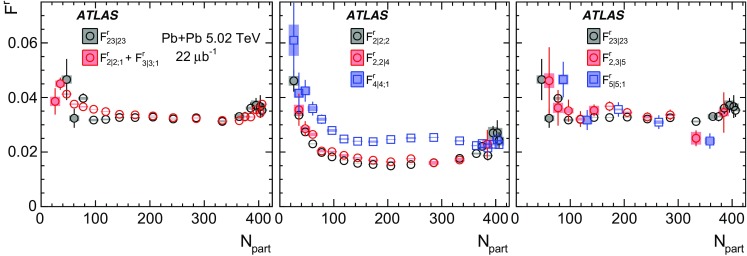
Comparison of the slopes of the correlators as a function of $N_{\mathrm {part}}$ for three groups of correlators: $r_{2,3|2,3}$ and $r_{2|2;1}r_{3|3;1}$ (for which the slope is $F_{2|2;1}+F_{3|3;1}$) in Fig. 21 (left panel), $r_{2|2;2}$, $r_{2,2|4}$ and $r_{4|4;1}$ in Fig. 22 (middle panel), and $r_{2,3|2,3}$, $r_{2,3|5}$ and $r_{5|5;1}$ in Fig. 23 (right panel). The error bars and shaded boxes are statistical and systematic uncertainties, respectively

The results are summarised in Fig. 24, with each panel corresponding to the slopes of distributions in Figs. 21, 22, and 23, respectively. The only significant difference is seen between $F_{4|4;1}$ and $F_{2|2;2}$ or $F_{2,2|4}$.

## Summary

Measurements of longitudinal flow correlations for charged particles are presented in the pseudorapidity range $|\eta |<2.4$ using 7 and 470 $\upmu \hbox {b}^{-1}$ of Pb+Pb data at $\sqrt{s_{\mathrm {NN}}}=2.76$ and 5.02 TeV, respectively, recorded by the ATLAS detector at the LHC. The factorisation of two-particle azimuthal correlations into single-particle flow harmonics $v_n$ is found to be broken, and the amount of factorisation breakdown increases approximately linearly as a function of the $\eta$ separation between the two particles. The slope of this dependence is nearly independent of centrality and $p_{\text {T}}\,$ for $n>2$. However, for $n=2$ the effect is smallest in mid-central collisions and increases toward more central or more peripheral collisions, and in central collisions the effect also depends strongly on $p_{\text {T}}\,$. Furthermore, the effect is found to be larger at 2.76 than 5.02 TeV for all harmonics, which cannot be explained entirely by the change in the beam rapidity.

The higher moments of the $\eta$-dependent flow correlations are also measured and the corresponding linear coefficients of the $\eta$ dependence are extracted. The coefficient for the $k{\text {th}}$-moment of $v_n$ scales with *k* for $n>2$, but scales faster than *k* for $n=2$. The factorisation breakdown is separated into contributions from forward-backward asymmetry of the flow magnitude and event-plane twist, which are found to be comparable to each other.

The longitudinal flow correlations are also measured between harmonic flows of different order. The correlation of $v_2v_3$ between two $\eta$ ranges is found to factorise into the product of the correlation for $v_2$ and the correlation for $v_3$, suggesting that the longitudinal fluctuations of $v_2$ and $v_3$ are independent of each other. The correlations between $v_4$ and $v_2^2$ suggest that the longitudinal fluctuations of $v_4$ have a significant nonlinear contribution from $v_2$, i.e. $v_4\propto v_2^2$. Similarly, the correlations between $v_5$ and $v_2v_3$ suggest that the longitudinal fluctuations of $v_5$ are driven by the nonlinear contribution from $v_2v_3$, i.e. $v_5\propto v_2v_3$. The results presented in this paper provide new insights into the fluctuations and correlations of harmonic flow in the longitudinal direction, which can be used to improve full three-dimensional viscous hydrodynamic models.

## Figures and Tables

**Fig. 25:**
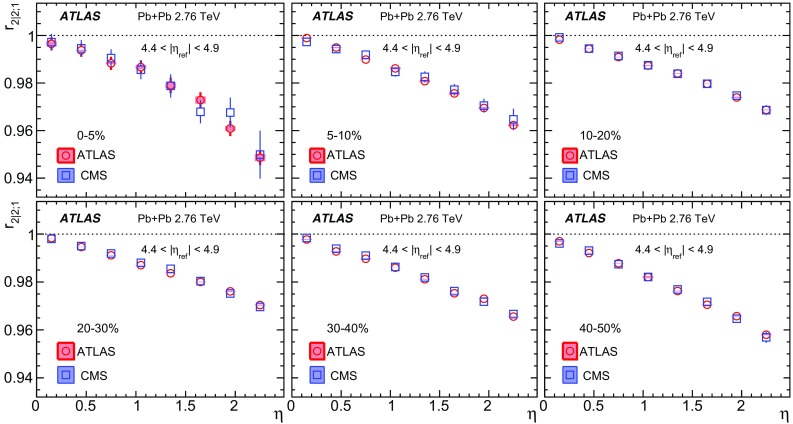
The values of $r_{2|2;1}$ measured by ATLAS and by CMS [19] for Pb+Pb collisions at 2.76 TeV, for the same reference pseudorapidity $4.4< |\eta _{\mathrm {ref}}|<4.9$

**Fig. 26:**
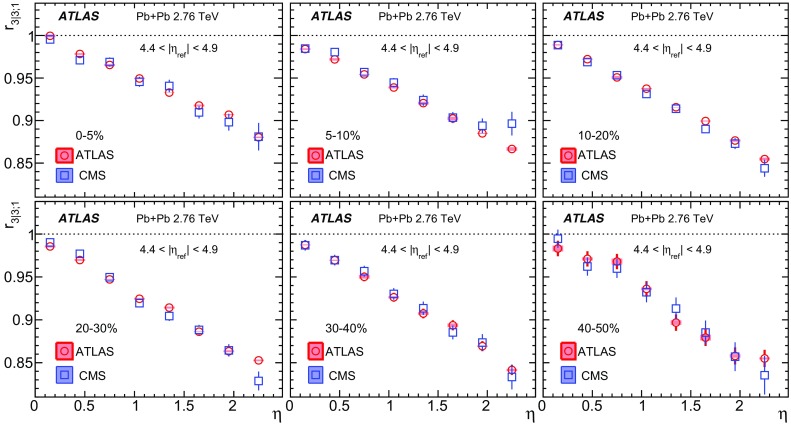
The values of $r_{3|3;1}$ measured by ATLAS and by CMS [19] for Pb+Pb collisions at 2.76 TeV, for the same reference pseudorapidity $4.4< |\eta _{\mathrm {ref}}|<4.9$

**Fig. 27:**
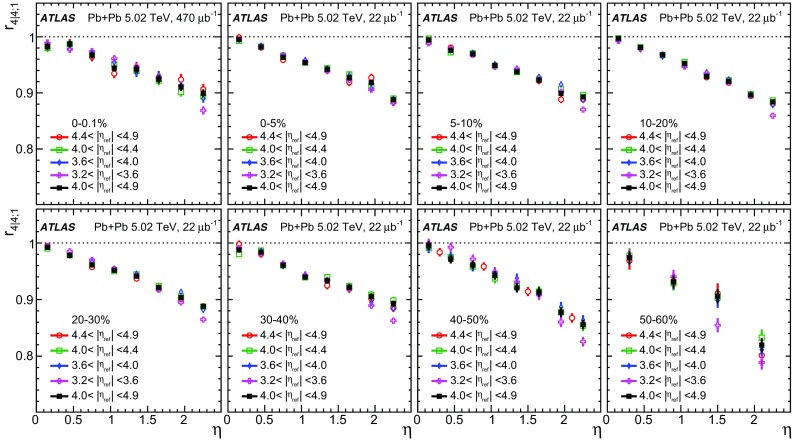
The $r_{4|4;1}(\eta )$ measured for several $\eta _{\mathrm {ref}}$ ranges for Pb+Pb collisions at 5.02 TeV. Each panel represents one centrality range. The error bars are statistical only. The data points in some centrality intervals are rebinned to reduce the uncertainty

**Fig. 28:**
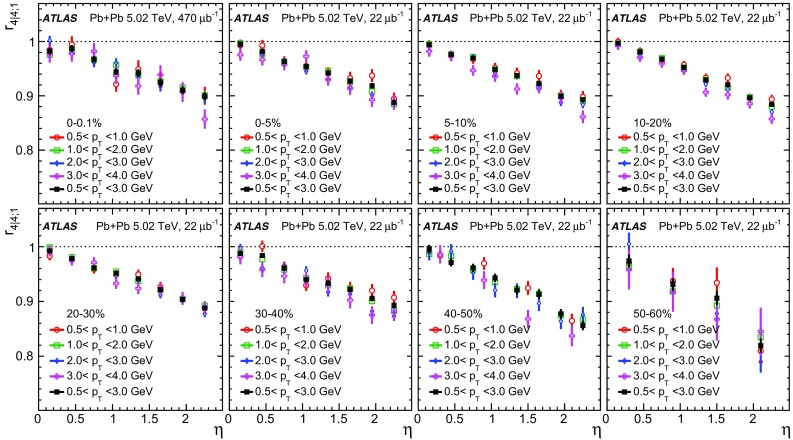
The $r_{4|4;1}(\eta )$ measured in several $p_{\text {T}}\,$ ranges for Pb+Pb collisions at 5.02 TeV. Each panel shows the results for one centrality range. The error bars are statistical only. The data points in some centrality intervals are rebinned to reduce the uncertainty

**Fig. 29:**
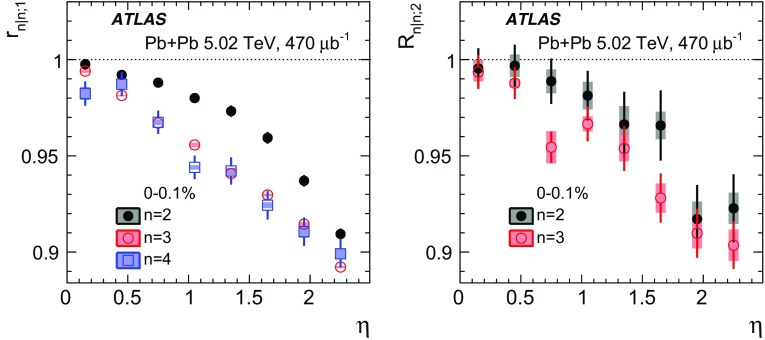
The $r_{n|n;1}(\eta )$ (left panel) and $R_{n|n;2}(\eta )$ (right panel) in ultra-central Pb+Pb collisions at 5.02 TeV. The error bars and shaded boxes are statistical and systematic uncertainties, respectively

## Appendix

Figures 25 and 26 show a comparison of $r_{2|2;1}$ and $r_{3|3;1}$ between ATLAS and CMS for Pb+Pb collisions at 2.76 TeV, where the ATLAS $\eta _{\mathrm {ref}}$ is chosen to be $4.4<|\eta _{\mathrm {ref}}|<4.9$ to match that of the CMS Collaboration. Excellent agreement is observed. Figure 27 and 28 show the detailed $p_{\text {T}}\,$ and $\eta _{\mathrm {ref}}$ dependence of $r_{4|4;1}$; these figures complement Figs. 2, 3, 4 and 5. Figure 29 compiles the results of $r_{n|n;1}$ and $R_{n|n;2}$ for 0–0.1% ultra-central collisions.

## Footnotes

1 As in several previous analyses [26, 27], a complex number is used to represent the real two-dimensional flow vector.

2 ATLAS uses a right-handed coordinate system with its origin at the nominal interaction point (IP) in the centre of the detector and the *z*-axis along the beam pipe. The *x* axis points from the IP to the centre of the LHC ring, and the *y* axis points upward. Cylindrical coordinates $(r,\phi )$ are used in the transverse plane, $\phi$ being the azimuthal angle around the beam pipe. The pseudorapidity is defined in terms of the polar angle $\theta$ as $\eta =-\ln \tan (\theta /2)$.
